# Supplementary material for: NeuroGT: A brain atlas of neurogenic tagging CreER drivers for birthdate-based classification and manipulation of mouse neurons
Source: Cell Rep Methods. 2021 May 25;1(3):100012. doi: 10.1016/j.crmeth.2021.100012 (PMC9017123; doi:10.1016/j.crmeth.2021.100012)
Supplement: Document S1. Figures S1 and S2 [file mmc1.pdf]

Cell Reports Methods, Volume *1*

Supplemental information

NeuroGT: A brain atlas of neurogenic tagging CreER drivers for birthdate-based classification and manipulation of mouse neurons

Tatsumi Hirata, Yukako Tohsato, Hiroya Itoga, Go Shioi, Hiroshi Kiyonari, Sanae Oka, Toshihiko Fujimori, and Shuichi Onami

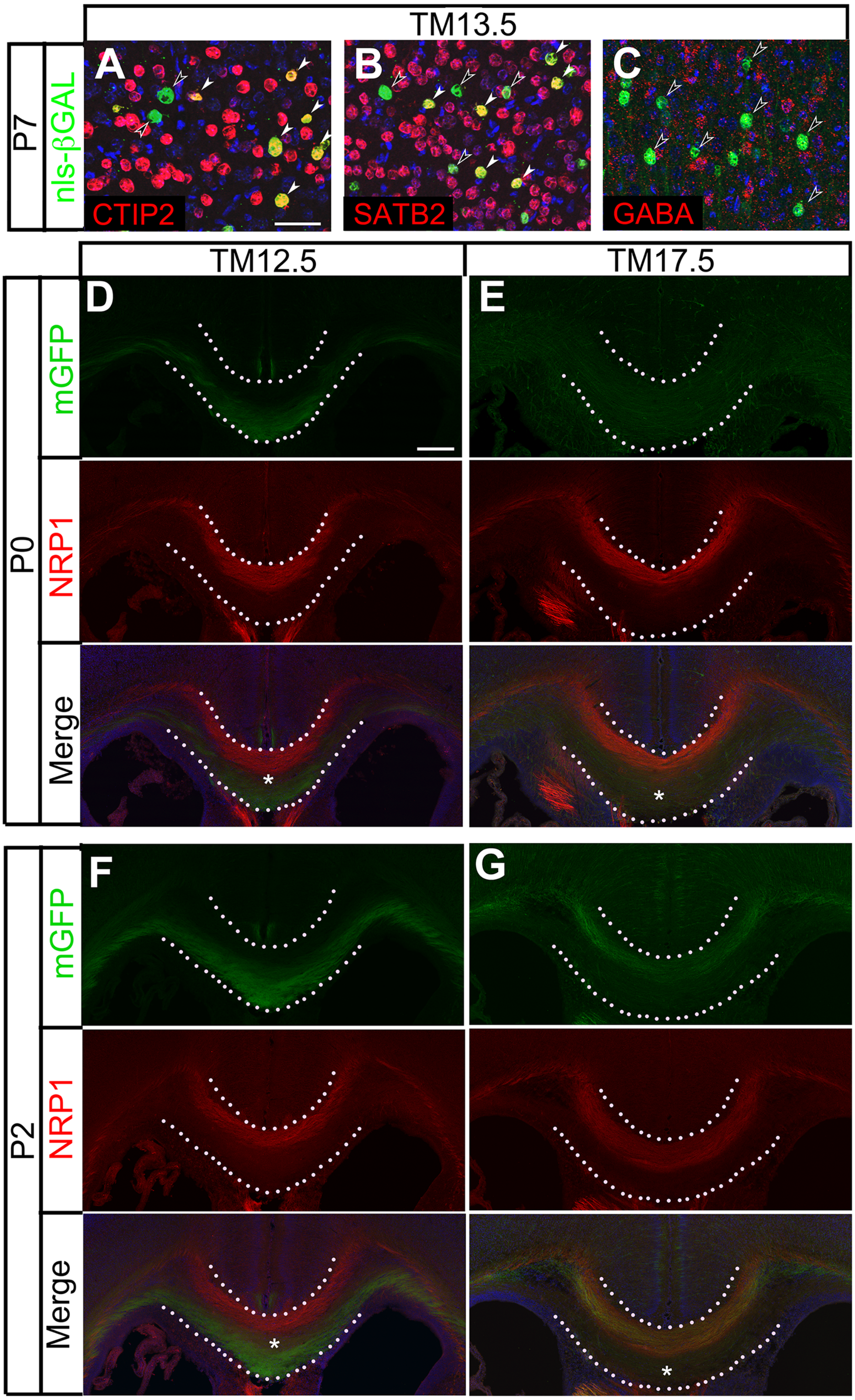

Figure S1. Cortical neurons and callosal projections tagged by Neurog2^{CreER}(G2A), Related to Figure 4
(A–C) Double immunostaining of TM13.5 cortical neurons in layer V for nls-βGAL (green) and anti-CTIP2 (red in A), anti-SATB2 (red in B) or anti-GABA (red in C) antibodies, and counterstained with DAPI (blue). Some tagged neurons express the subcortically projecting neuron marker CTIP2 (filled arrowheads in A) or callosally projecting neuron marker SATB2 (filled arrowheads in B), but not the inhibitory interneuron marker GABA (C). Open arrowheads indicate tagged neurons negative for these markers. (D–G) Corpus callosum sections (16-μm thick) obtained from P0 (D, E) and P2 (F, G) mice that were tagged at TM12.5 (D, F) or TM17.5 (E, G). Dotted lines delineate the corpus callosum. mGFP (green), anti-NRP1 (red) and their merged images counterstained with DAPI (blue). NRP1 marks the dorsal callosal bundle projected from the medial cortical areas only in the embryonic and neonatal stages. TM12.5-tagged axons course through the ventral edge of the callosum both at P0 (D) and P2 (F), leaving a small open space (asterisk) from the NRP1-positive bundle. TM17.5-tagged axons seem to still elongate during these neonatal stages; the axons are widely distributed across the whole callosum at P0 (E), whereas they are more concentrated in the dorsal zone marked by NRP1 and sparsely scattered in the ventral zone (asterisk) at P2 (G). For visualization of the TM17.5-tagged axons (E, G), an anti-GFP antibody was used because mGFP fluorescence was still weak at the neonatal stages. Bar= 200 μm.

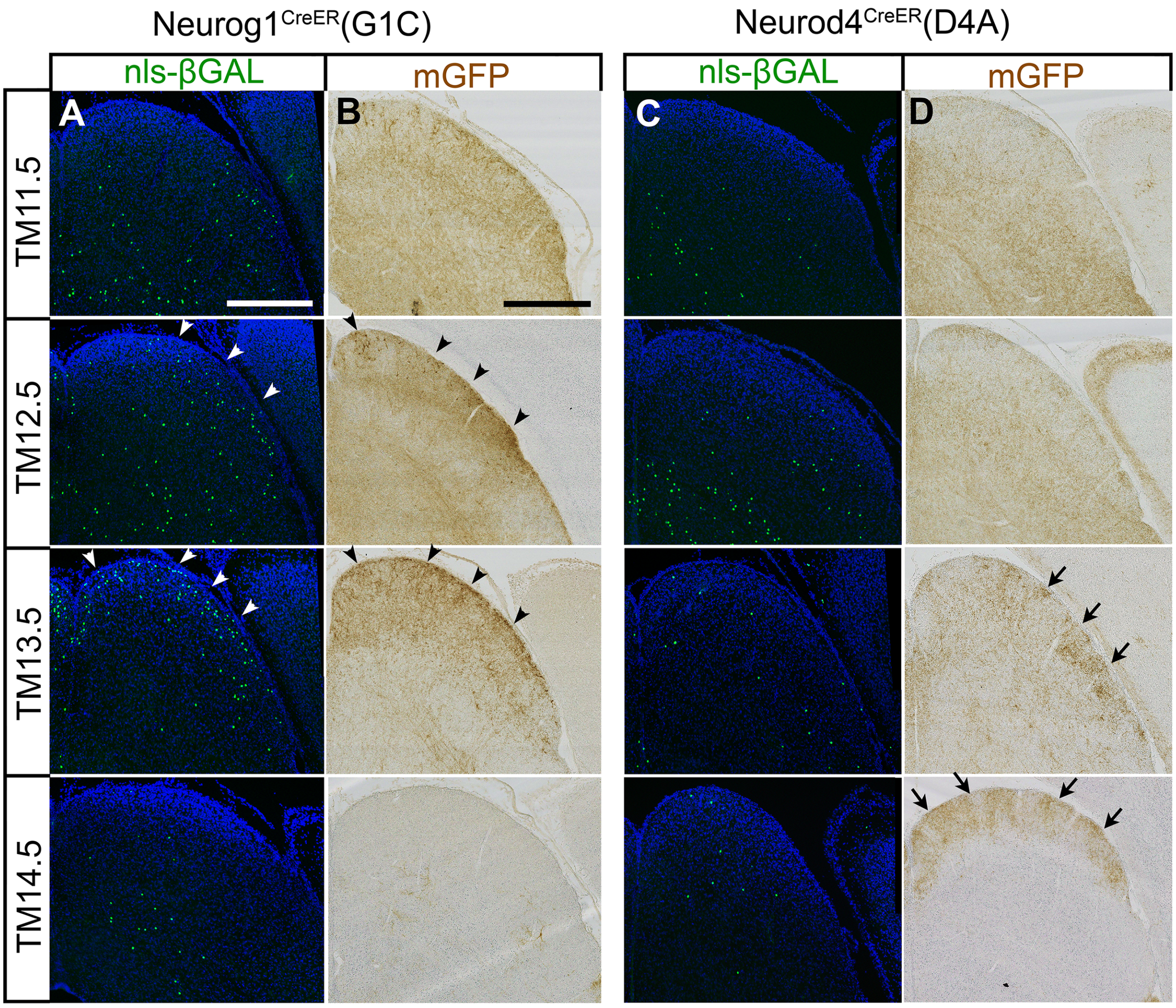

Figure S2. Superior colliculus tagged by Neurog1^{CreER}(G1C) and Neurod4^{CreER}(D4A), Related to Figure 6
(A–D) nls-βGAL (A, C) and mGFP (B, D) staining of superior colliculus sections obtained from P7 mice tagged by Neurog1^{CreER}(G1C) (A, B) and Neurod4^{CreER}(D4A) (C, D) drivers at different stages indicated on the left. Neurons in the superficial sensory layers (arrowheads) are labeled at TM12.5-13.5 by Neurog1^{CreER}(G1C) driver (A, B). Arrows in (D) indicate tagged retinal axons that target the superficial layers. Bar= 500 µm.
