# Supplementary material for: NeuroGT: A brain atlas of neurogenic tagging CreER drivers for birthdate-based classification and manipulation of mouse neurons
Source: Cell Rep Methods. 2021 May 25;1(3):100012. doi: 10.1016/j.crmeth.2021.100012 (PMC9017123; doi:10.1016/j.crmeth.2021.100012)
Supplement: Document S2. Article plus supplemental information [file mmc3.pdf]

Cell Reports
Methods

Article

NeuroGT: A brain atlas of neurogenic tagging CreER drivers for birthdate-based classification and manipulation of mouse neurons

Graphical abstract

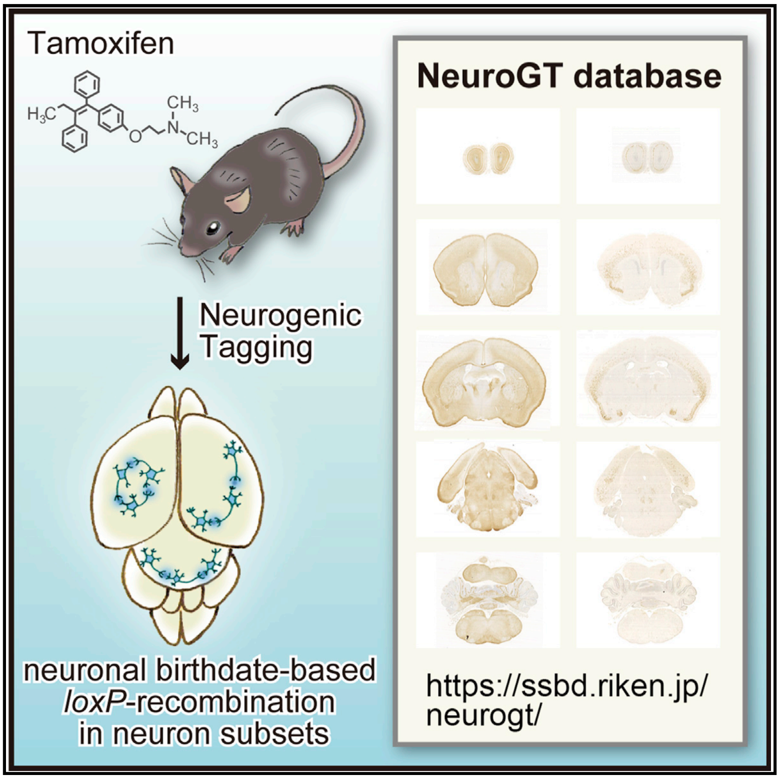

Authors

Tatsumi Hirata, Yukako Tohsato, Hiroya Itoga, ..., Sanae Oka, Toshihiko Fujimori, Shuichi Onami

Correspondence

tathirat@nig.ac.jp

In brief

Hirata et al. develop neurogenic tagging CreER driver mice and the NeuroGT database to showcase the mouse lines. The resource can be used to classify neurons on the basis of their generation timing and manipulate the classified neuron subsets for research purposes.

Highlights

- We generate four CreER mouse drivers to tag neurons on the basis of time of neurogenesis
- The drivers enable birthdate-based classification and manipulation of neurons
- We develop NeuroGT, an image atlas of tagged neurons across the entire brain
- Through NeuroGT, researchers can find driver lines suitable for their own research

Hirata et al., 2021, Cell Reports Methods *1*, 100012
July 26, 2021 © 2021 The Author(s).
https://doi.org/10.1016/j.crmeth.2021.100012

CellPress

Cell Reports Methods

CellPress
OPEN ACCESS

Article

NeuroGT: A brain atlas of neurogenic tagging CreER drivers for birthdate-based classification and manipulation of mouse neurons

Tatsumi Hirata,[1,2,8,*] Yukako Tohsato,[3,4] Hiroya Itoga,[4] Go Shioi,[5] Hiroshi Kiyonari,[5] Sanae Oka,[6] Toshihiko Fujimori,[2,6] and Shuichi Onami[4,7]

[1]Brain Function Laboratory, National Institute of Genetics, 1111 Yata, Mishima 411-8540, Japan
[2]The Graduate University for Advanced Studies, SOKENDAI, Hayama, Japan
[3]Computational Biology Laboratory, Faculty of Information Science and Engineering, Ritsumeikan University, 1-1-1 Noji-higashi, Kusatsu, Shiga 525-8577, Japan
[4]Laboratory for Developmental Dynamics, RIKEN Center for Biosystems Dynamics Research, 2-2-3 Minatojima-minamimachi, Chuo-ku, Kobe 650-0047, Japan
[5]Laboratory for Animal Resources and Genetic Engineering, RIKEN Center for Biosystems Dynamics Research, 2-2-3 Minatojima-minamimachi, Chuo-ku, Kobe 650-0047, Japan
[6]Division of Embryology, National Institute for Basic Biology, 5-1 Higashiyama, Myodaiji, Okazaki 444-8787, Japan
[7]Life Science Data Sharing Unit, RIKEN Information R&D and Strategy Headquarters, 2-2-3 Minatojima-minamimachi, Chuo-ku, Kobe 650-0047, Japan
[8]Lead contact
*Correspondence: tathirat@nig.ac.jp
https://doi.org/10.1016/j.crmeth.2021.100012

MOTIVATION Neuronal birthdates are commonly used to classify neurons. However, the current nucleotide-based birthdating method is retrospective in nature and does not allow the manipulation of classified neurons. To overcome this limitation, we developed neurogenic tagging mouse lines that can induce CreER-*loxP* recombination in neurons with similar birthdates, and the recombination tag can be subsequently used for experimental manipulation of the classified neuron subsets. To encourage the use of this resource, we launched the NeuroGT database, which contains image data of the tagged neurons across the entire brain.

SUMMARY

Neuronal birthdate is one of the major determinants of neuronal phenotypes. However, most birthdating methods are retrospective in nature, allowing very little experimental access to the classified neuronal subsets. Here, we introduce four neurogenic tagging mouse lines, which can assign CreER-*loxP* recombination to neuron subsets that share the same differentiation timing in living animals and enable various experimental manipulations of the classified subsets. We constructed a brain atlas of the neurogenic tagging mouse lines (NeuroGT), which includes holistic image data of the *loxP*-recombined neurons and their processes across the entire brain that were tagged on each single day during the neurodevelopmental period. This image database, which is open to the public, offers investigators the opportunity to find specific neurogenic tagging driver lines and the stages of tagging appropriate for their own research purposes.

INTRODUCTION

During development, neurons are generated over a protracted time window. Mounting evidence shows that neurogenic timing, which is often referred to as the neuronal birthdate, has immense impacts on neuronal phenotypes. In the neocortex, the birthdate determines layer positioning, connection patterns, and molecular and physiological properties of neurons (McConnell, 1989; Lodato and Arlotta, 2015). The chronological specification of neuronal fates is not a unique property of the neocortex but rather a generally conserved strategy in various nervous systems for generating neuronal diversity (Suzuki and Hirata, 2013). Even without affecting the intrinsic molecular differences, the birth timing itself could cause differential neuronal phenotypes because neurons with different birthdates are influenced by a changing environment (Hirata and Iwai, 2019). Because of its intimate relationships with various neuronal phenotypes, neuronal birthdating has been a reliable standard for the classification of

This is an open access article under the CC BY license (http://creativecommons.org/licenses/by/4.0/).

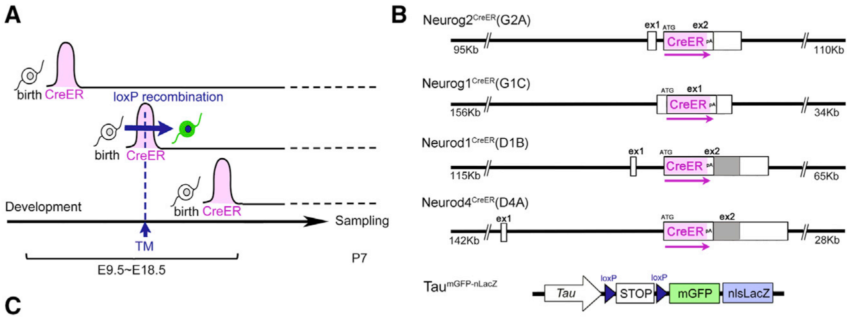

C

Line Name	Code	Enhancer	Notable regions tagged	Deposited number
Neurog2^{CreER}	G2A	neurogenin2	olfactory bulb, piriform cortex, neocortex, amygdala, hippocampus, mammillary body, inferior colliculus, cerebellum, olive, spinal cord	CDB0512T−1
Neurog1^{CreER}	G1C	neurogenin1	superior colliculus, inferior colliculus, pontine nucleus, cochlear nucleus	CDB0526T
Neurod1^{CreER}	D1B	neuroD1	subplate neuron, medial cortex, amygdala, hippocampus, retina, habenula, inferior colliculus, cochlear nucleus, sensory ganglia	CDB0510T
Neurod4^{CreER}	D4A	neuroD4	hippocampus, retina, habenula, supramammilary nucleus, inferior colliculus, cochlear nucleus, olive, spinal cord, peripheral ganglia	CDB0511T

Figure 1. Neurogenic tagging and the driver mouse lines

(A) In neurogenic tagging mouse lines, CreER is transiently expressed within a short neurogenic time window in various neurons. TM injection on a single day during E9.5–E18.5 induces *loxP* recombination only in cells expressing CreER. The mouse embryos were raised up to P7, and the brains were sampled for visualization of tagged neurons.

(B) The BAC genomic constructs recombined with the CreER cassette that were used to generate the four neurogenic tagging driver lines. Boxes show the exons, in which the shaded part represents originally protein-coding sequences and the white part untranslated sequences. The approximate lengths of the genome upstream and downstream of the CreER cassette are indicated under each construct. The bottom panel shows a schematic of the gene structure of the Tau$^{mGFP-nLacZ}$ reporter. Both reporter proteins, mGFP and nls-βGAL, were used to visualize tagged neurons.

(C) Four neurogenic tagging mouse lines were characterized in this study. The mice can be obtained from RIKEN BDR (http://www2.clst.riken.jp/arg/TG%20mutant%20mice%20list.html).

neurons in various nervous systems for over half a century (Bayer and Altman, 1987; Govindan et al., 2018).

Traditionally, neuronal birthdating has been performed by using nucleotide analogs, such as radioactive thymidine and, more recently, bromodeoxyuridine (BrdU) or 5-ethyynyl uridine (EdU), which are incorporated into the DNA during the S phase to mark the final round of the cell cycle. Although these techniques have substantially improved, they are still descriptive histological techniques to determine neuronal birthdates in sacrificed animals. Recently, we developed a neurogenic tagging method by which tamoxifen (TM)-dependent CreER (Feil et al., 1997) recombination was induced in olfactory bulb neurons in a birthdate-dependent manner (Hirata et al., 2019). Given that this technique is based on irreversible *loxP* recombination, the tag can be used later to explore the birthdate-classified neuronal subset in various ways.

This neurogenic tagging method uses driver mouse lines in which CreER is expressed only transiently within a short time window immediately after neuronal fates are committed (Figure 1A). Consequently, a single administration of TM at a certain developmental stage induces the recombination of *loxP* sequences only in the cells that express CreER. In the previous study, we used the enhancer of the *neurog2* gene to achieve the transient expression of CreER in a bacterial artificial chromosome (BAC) transgenic mouse. Although the biological principle underlying this method differs from that of nucleotide-based birthdating, the CreER driver that we developed achieved birthdate-dependent neuron tagging in the brain regions such as the olfactory bulb (Hirata et al., 2019) and cerebellum (Tran-Anh et al., 2020; Zhang et al., 2020), mirroring the endogenous expression patterns of *neurog2*.

To cover other brain regions, we developed three neurogenic tagging driver lines by using the neuronal differentiation genes *neurog1*, *neurod1*, and *neurod4* (Figures 1B and 1C), all of which are basic-helix-loop-helix transcription factors that are transiently expressed during the maturation phase of neurons (Kim et al., 2011; Sudarov et al., 2011; Aprea et al., 2014). Together with the previous line developed by using *neurog2*, the collection of driver lines covers most of the mouse nervous systems. Our aim is to contribute this resource to the scientific community. To encourage the use of this resource, we have developed a brain atlas of neurogenic tagging mouse lines (NeuroGT). This database contains section images of the entire brain visualized for tagged neurons as well as their processes; these images were obtained from postnatal mice that received a single TM injection on each day during the neurogenetic period. Researchers interested in particular brain regions can find appropriate mouse lines and tagging stages for their research purposes.

RESULTS

NeuroGT and neurogenic tagging driver mice

The neurogenic tagging drivers Neurog2^{CreER}(G2A), Neurog1^{CreER}(G1C), Neurod1^{CreER}(D1B), and Neurod4^{CreER}(D4A) were developed as described in STAR Methods and are deposited at the RIKEN Center for Biosystems Dynamics Research (RIKEN BDR) (Figures 1B and 1C). To showcase the tagged neuron images comprehensively, each neurogenic tagging line was crossed with Tau$^{mGFP-nLacZ}$ mouse (Hippenmeyer et al., 2005), which is a global neuronal Cre reporter that expresses dual nucleus- and membrane-localized reporters under a constitutive neuronal promoter after the excision of the *loxP-STOP-*

loxP cassette (Figure 1B). Staged pregnant mice were then intraperitoneally injected with TM only once during the gestation stages at embryonic day 9.5 (E9.5)–E18.5 (hereafter called TM9.5–TM18.5, referring to TM injection stages), and the tagged offspring were delivered and raised up to postnatal day 7 (P7).

Figure 2 shows X-gal-stained images of whole-mount brains tagged by the four drivers at different TM stages. The tagged neurons stained blue by the nucleus-localized (nls)-βGAL reporter were distributed in different brain regions depending on the TM injection stages and on the driver lines. Some brain regions were tagged by multiple drivers, whereas others were tagged specifically only by a single driver (Figure 2 and Table 1). From the overall external appearance, a neurogenic wave was observed to migrate from posterior regions, such as the hindbrain, to the more anterior regions according to the TM injection stages (Figure 2). In the brain regions tagged by multiple drivers, the spatiotemporal patterns of tagging were basically similar (Table 1), indicating that all the driver lines capture a similar neurogenetic time window in neurons.

To expose the internal structures, we collected and coronally sectioned brains tagged at different TM stages by individual drivers. The interspaced serial sections were antibody stained with diaminobenzidine (DAB) for either the nls-βGAL or the membrane-localized mGFP reporter. All these sections were converted into high-resolution digital images (e.g., Figures 6B–6D) and subgrouped into datasets according to three categories, namely the driver line, TM stage, and stained reporter (nls-βGAL or mGFP). Each dataset defined by the combination of the three categories contained 142–180 interspaced section images, which were aligned along the entire anterocaudal axis of the brain (approximately 1.2 mm in length). This coverage is sufficiently high to identify even a small nucleus or structure in the brain (Table 1).

All datasets of the high-resolution section images (835 GB of data for 13,538 images in 84 datasets including TM-negative controls) and whole-mount images shown in Figure 2 are available in the NeuroGT database (https://ssbd.riken.jp/neurogt/). Users can search for these datasets from a web browser by using terms that match the meta-information stored in the database, such as their identifier, driver name, TM stage, or reporter used for staining (Figure 3A). The search results are displayed as a list of links to the dataset page (Figure 3B). To enable this search and visualization, development of the NeuroGT was based on the SSBD (Systems Science of Biological Dynamics) database (Tohsato et al., 2016). The dataset pages at the next stage allow users to download the high-resolution section images (Figure 3C) and interactively view their thumbnails together with the meta-information. The thumbnail images of nls-βGAL- and mGFP-stained sections are stacked separately in order along the anteroposterior axis (Figure 3D) and can be viewed sequentially by dragging the slider (Video S1). The search function for anatomical regions will be implemented in the future. Through NeuroGT, researchers can identify the drivers useful for their research and obtain them from RIKEN BDR (http://www2.clst.riken.jp/arg/TG%20mutant%20mice%20list.html).

Table 1 summarizes the brain regions that were tagged by individual drivers. When the tagged stages were compared with the reported birthdates determined in mice by using nucleotide analogs, the ranges were fairly consistent (Table 1). The exceptions might be the pontine nucleus and dorsal root ganglion. Considering the short time lag often observed between the final S phase and the expression of CreER (Florio et al., 2012; Toma et al., 2014; Hirata et al., 2019; see also this paper), the TM stages for labeling these regions slightly preceded the birthdates determined with nucleotide analogs, suggesting that the *loxP* recombination might have occurred in pre-mitotic neuronal progenitors in these regions. Table 1 lists only limited brain regions from areas containing extensive tagged neurons. The NeuroGT database can be used to access and visualize a specific brain region of interest.

The following sections describe the features of each driver line, focusing on specific regions that can be effectively tagged by a particular line.

Neurog2^{CreER}(G2A) driver using the *neurog2* enhancer

The Neurog2^{CreER}(G2A) line tags the most extensive brain regions. Specifically, at TM10.5–TM12.5, sagittal stripes in the cerebellum were labeled (magenta arrowheads 1 in Figure 2A), reflecting birthdate-dependent generation of cerebellar Purkinje neurons (Hashimoto and Mikoshiba, 2003; Namba et al., 2011). TM injection between TM11.5 and TM17.5 heavily labeled olfactory bulb neurons, as reported previously (Hirata et al., 2019), and neurons in the central olfactory areas and the amygdala (blue arrowheads 2 in Figure 2A). The dense labeling of the piriform cortex at TM11.5–TM12.5 demarcated a sharp border from the neocortex that was labeled by later TM injections, as will be described in the next paragraph (see also Figure 4C). In the inferior colliculus of the midbrain, a neurogenic gradient from the lateral to the mediocaudal was marked at TM12.5–TM16.5 (brown arrowheads 3 in Figure 2A) as previously reported (Altman and Bayer, 1981). Labeling of the inferior colliculus was also observed in the same spatiotemporal pattern by other neurogenic tagging drivers (brown arrowheads 3 in Figures 2C and 2D; Table 1).

In cross-sections of the neocortex, the inside-out birth-order arrangement of neurons was clearly visible with the nls-βGAL reporter (Figure 4A). As expected from the *neurog2* expression, only excitatory projection neurons, but not GABAergic inhibitory neurons, were tagged in the neocortex (Figures S1A–S1C). The mGFP reporter prominently labeled neuronal fibers, including axons and dendrites (Figures 4B–4F). In a close-up of the corpus callosum (Figure 4B), long axons projecting from the tagged neocortical neurons were visualized. Interestingly, the axons of the early-born (TM12.5–TM13.5) and late-born (TM16.5–TM17.5) cortical neurons formed dorsoventrally segregated fascicles within the callosum, presenting a feature that would have been difficult to recognize by nucleotide-based birthdating. Previous studies have reported the dorsoventral segregation of callosal axons from the medial and lateral cortical areas (Piper et al., 2009; Nishikimi et al., 2011; Zhou et al., 2013). The present axon compartments formed by the early-born and late-born callosal neurons appeared to be different from those based on the distinct cortical areas (Figures S1D–S1G), adding complexity to the organization of the corpus callosum.

Another unique feature of the neocortex was the patchy areal distribution of superficial neurons in the layer II/III tagged around

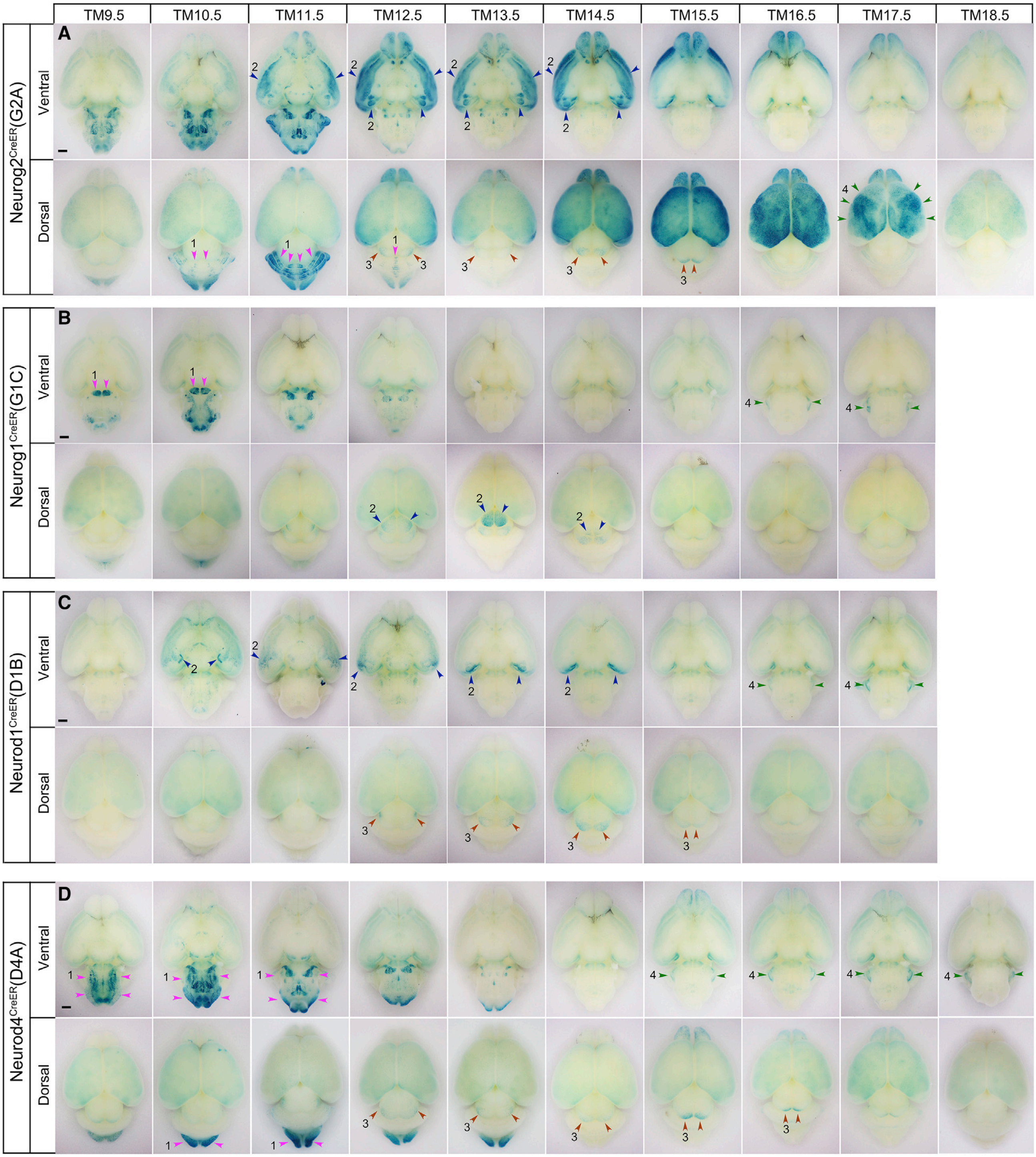

Figure 2. Neurogenic-tagged neurons in whole-mount brains

Ventral and dorsal views of P7 brains tagged by Neurog2^{CreER}(G2A) (A), Neurog1^{CreER}(G1C) (B), Neurod1^{CreER}(D1B) (C), and Neurod4^{CreER}(D4A) (D) drivers. The TM injection stages are indicated at the top. Magenta arrowheads 1 show cerebellar Purkinje stripes in (A), pontine nuclei in (B), and olive nuclei and other structures in the medulla in (D). Blue arrowheads 2 show the olfactory cortex and amygdala nuclei in (A) and (C), and the superior colliculus in (B). Brown arrowheads 3 show the inferior colliculus in (A), (C), and (D). Green arrowheads 4 show the area-specific distribution of superficial cortical neurons in (A), and cochlear nuclei in (B), (C), and (D). Scale bars, 1 mm.

Table 1. Comparison of neurogenic-tagged stages identified in current study and the birthdates determined in previous papers

Region	Neurogenic-tagged stage	Driver line	Tagged territory	Birthdate reported	Method	Reference
Olfactory bulb	TM11.5–TM17.5	Neurog2^{CreER}(G2A)	from deep to surface	E10.5–E17.5	$[^3H]$thymidine	Hinds, 1968
		Neurod4^{CreER}(D4A)				
Piriform cortex	TM11.5–TM14.5	Neurog2^{CreER}(G2A)		E12.5 (peak)	BrdU	Sarma et al., 2011
Amygdala	TM12.5–TM15.5	Neurog2^{CreER}(G2A)	distinct nuclei	E11.5–E15.5	$[^3H]$thymidine	McConnell and Angevine, 1983
		Neurod1^{CreER}(D1B)				
Neocortex	TM12.5–TM17.5	Neurog2^{CreER}(G2A)	from deep to surface	E11.5–E17.5	$[^3H]$thymidine	Caviness, 1982
Subplate neuron (neocortex)	TM11.5–TM12.5	Neurod1^{CreER}(D1B)		E11.5–E12.5	BrdU	Hoerder-Suabedissen and Molnar, 2013
Hippocampus pyramidal neuron	TM13.5–TM17.5	Neurog2^{CreER}(G2A)	from deep to surface	E12.5–E16.5	$[^3H]$thymidine	Caviness, 1973
		Neurod1^{CreER}(D1B)				
		Neurod4^{CreER}(D4A)				
Habenula	TM11.5–TM16.5	Neurog2^{CreER}(G2A)	from lateral to medial	E11.5–E16.5	$[^3H]$thymidine	Angevine, 1970
		Neurod1^{CreER}(D1B)				
		Neurod4^{CreER}(D4A)				
Mammillary body	TM11.5–TM16.5	Neurog2^{CreER}(G2A)	from lateral to medial	E9.5–E13.5	BrdU	Szabo et al., 2015
		Neurog1^{CreER}(G1C)				
Supramammillary nucleus	TM10.5–TM16.5	Neurod4^{CreER}(D4A)		E10.5–E14.5	$[^3H]$thymidine	Shimada and Nakamura, 1973
Superior colliculus	TM11.5–TM14.5	Neurog1^{CreER}(G1C)		E11.5–E14.5	$[^3H]$thymidine	Edwards et al., 1986
Inferior colliculus	TM12.5–TM16.5	Neurog2^{CreER}(G2A)	from lateral to medial	E12.5–E15.5	$[^3H]$thymidine	Zeng et al., 2009
		Neurog1^{CreER}(G1C)				
		Neurod1^{CreER}(D1B)				
		Neurod4^{CreER}(D4A)				
Cerebellum Purkinje cell	TM10.5–TM12.5	Neurog2^{CreER}(G2A)	sagittal stripes	E10.5–E12.5	BrdU	Hashimoto and Mikoshiba, 2003
Pontine nucleus	TM9.5–TM11.5	Neurog1^{CreER}(G1C)		E12.5–E14.5	BrdU	Kawauchi et al., 2006
Trapezoid body	TM11.5–TM12.5	Neurog2^{CreER}(G2A)		E11.5–E12.5	$[^3H]$thymidine	Pierce, 1973
		Neurog1^{CreER}(G1C)				
		Neurod4^{CreER}(D4A)				
Inferior olive	TM10.5–TM12.5	Neurog2^{CreER}(G2A)		E9.5–E11.5	$[^3H]$thymidine	Pierce, 1973
		Neurod4^{CreER}(D4A)				
Cochlear nucleus	TM15.5–TM17.5	Neurog1^{CreER}(G1C)		E14.5–E18.5 (second wave)	BrdU	Shepard et al., 2019
		Neurod1^{CreER}(D1B)				
		Neurod4^{CreER}(D4A)				
Spinal cord	TM9.5–TM13.5	Neurog2^{CreER}(G2A)	from ventral to dorsal	E9.5–E13.5	$[^3H]$thymidine	Nornes and Carry, 1978
		Neurod4^{CreER}(D4A)				
Dorsal root ganglion	TM9.5–TM12.5	Neurod1^{CreER}(D1B)		E10.5–E13.5	$[^3H]$thymidine	Lawson and Biscoe, 1979
		Neurod4^{CreER}(D4A)				

Representative brain regions are tagged by the neurogenic tagging driver lines. The TM stages for tagging are compared with the birthdates determined by using nucleotide analogs in mice. The reported birthdates are adjusted to set the day of mating at E0.5.

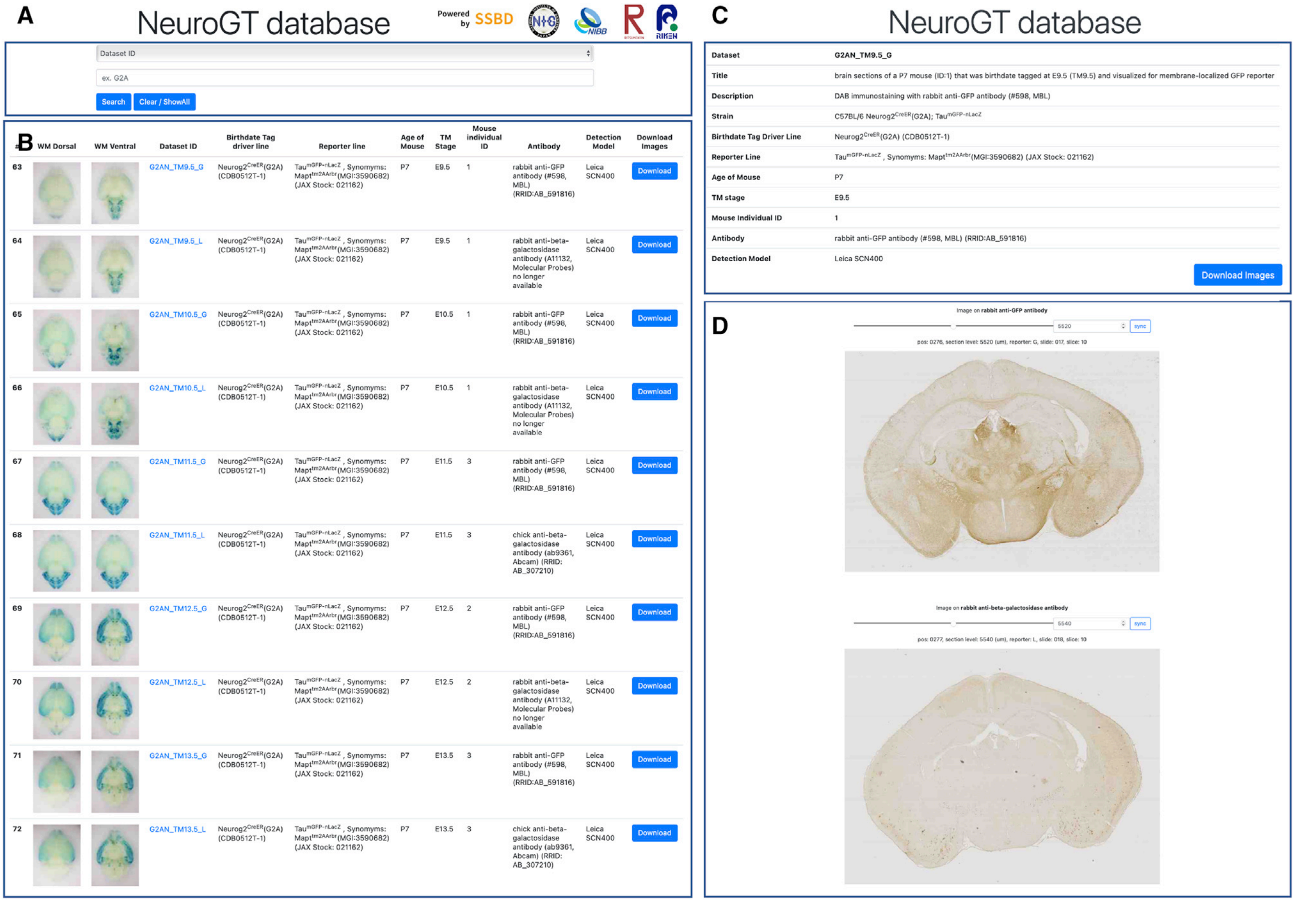

Figure 3. Screenshots from NeuroGT database

(A) Input window for keyword search.

(B) An example of a search result, which is returned as links to individual dataset pages with whole-mount brain images and selected meta-information.

(C) Meta-information of high-resolution section images and a button that allows users to download the images.

(D) Interactive viewer of the image thumbnails. The images of nls-βGAL and mGFP are stacked separately. By dragging the slider, coronal sections can be viewed sequentially along the anteroposterior axis. The sync button automatically matches the section level of the two reporter images. See also Video S1.

the late TM17.5 stage (green arrowheads 4 in Figure 2A). Somatosensory and visual areas contained packed X-gal-labeled neurons, whereas frontal, motor, and medial cingulate areas contained less abundant labeled neurons. The biased areal distribution of these superficial neurons was also confirmed in neocortical sections (Figures 4F and 4F′). A previous nucleotide-based neuronal birthdating study only reported a neurogenetic gradient in the superficial layers (Bayer and Altman, 1991). Perhaps the discrete mosaic distribution of superficial neurons was more readily recognizable in a whole-mount cortical representation by using neurogenic tagging.

In the hippocampus, pyramidal neurons in the CA1–CA3 regions were labeled with embryonic TM injections (Figure 5A). Interestingly, deep and superficial neurons in the CA1 layer were separately labeled by different TM injection times (Figure 5A′). They appear to be the neuronal subsets in the radial axis that have recently attracted much attention (Slomianka et al., 2011; Soltesz and Losonczy, 2018). The membrane-localized mGFP reporter, on the other hand, visualized the laminar organization of the hippocampus (Forster et al., 2006), where laminar-specific afferent axons from other brain regions and local projections of hippocampal proper neurons were differentially labeled depending on the TM stage (Figure 5B).

To characterize the timing of individual neurons that underwent TM-induced recombination in relation to their last cell cycle, we conducted a double injection of TM at the fixed TM12.5 stage and of EdU at a certain time point either before or after TM injection (Figures 5C–5F). Although the time courses for the emergence of double-positive neurons for EdU and the nls-βGAL reporter varied across brain regions, in several brain domains such as the piriform cortex (Figure 5C), mammillary body (Figure 5D), and cerebellum (Figure 5E), TM-induced recombination was most prevalent in neurons 6–12 h after the final DNA synthesis. This time course was similar to that previously observed in olfactory bulb neurons (Hirata et al., 2019). There were, however, a few exceptions; for example, neurons in the trapezoid body were most frequently double labeled when EdU and TM were co-injected (Figure 5F), suggesting that TM-induced recombination occurs

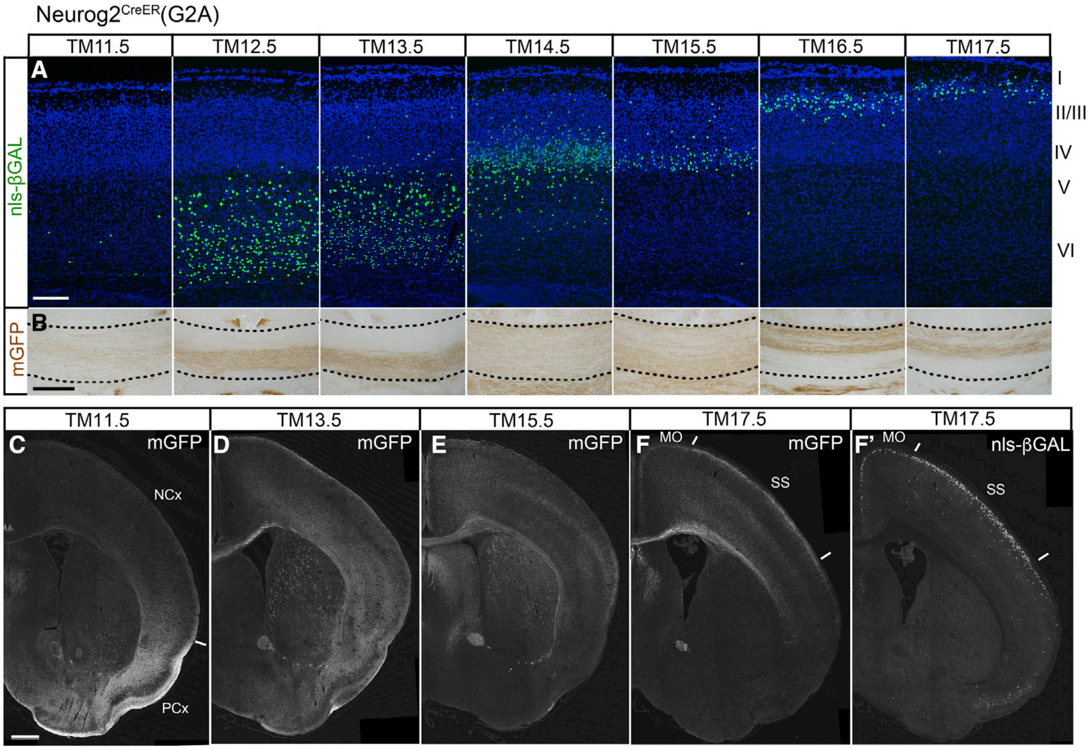

Figure 4. Cortex and telencephalic hemispheres tagged by Neurog2^{CreER}(G2A)

(A) P7 cortical sections stained for nls-βGAL. The tagged neurons with green-stained nuclei are arranged inside-out across the cortical layers according to the TM injection stages shown at the top.

(B) P7 corpus callosum (between dotted lines) stained for mGFP. Note that axons tagged at TM12.5–TM13.5 and TM16.5–TM17.5 course through the ventral and dorsal parts of the callosum, respectively. See also Figure S1.

(C–F) mGFP staining of telencephalic sections from P7 mice tagged at TM11.5 (C), TM13.5 (D), TM15.5 (E), and TM17.5 (F). Different fiber systems and neurons are visualized depending on the stage of TM injection. (F′) A section stained for nls-βGAL from the same mouse as that in (F). The white line in (C) indicates the boundary between the neocortex (NCx) and the piriform cortex (PCx). In (F) and (F′), tagged neurons are more abundant in the somatosensory area (SS) than in the motor area (MO).

Scale bars, 200 μm (A and B) and 500 μm (C–F).

in the progenitor stage before the final DNA synthesis. Nonetheless, a peak of double-positive neurons was detected in a specific time interval between EdU and TM injections, implying that the TM-induced recombination marks the neurons only within a short differentiation time window (Figure 5F).

Neurog1^{CreER}(G1C) driver using the *neurog1* enhancer

The characteristic regions tagged by the Neurog1^{CreER}(G1C) driver were the pontine nucleus (magenta arrowheads 1 in Figure 2B) and the superior colliculus in the midbrain (blue arrowheads 2 in Figure 2B), both of which were not labeled in whole-mount brain preparations by the other three drivers (Figures 2A–2D and Table 1).

In sections of the superior colliculus, neurons in the superficial sensory layers that receive retinal axons were labeled at TM12.5–TM13.5, and neurons in the deep motor layers were labeled sparsely for a more protracted TM11.5–TM14.5 (Figures S2A and S2B). This spatiotemporal pattern of labeling resembles the reported pattern of neurogenesis in the superior colliculus (Edwards et al., 1986), where superficial and deep-layer neurons are generated as distinct compartments following different time courses. Scattered neurons in the deep motor layers were also tagged by the other drivers at the same TM11.5–TM14.5 stages (Figures S2C and S2D), although they were invisible in the whole-mount preparations (Figure 2). The observation that the superficial layer neurons were only tagged by the Neurog1^{CreER}(G1C) driver seems to be consistent with the idea that the superficial sensory layers and the deep motor layers of the superior colliculus are populated with neuronal populations of distinct origins.

Neurod1^{CreER}(D1B) driver using the *neurod1* enhancer

The Neurod1^{CreER}(D1B) driver tagged only several externally visible structures in the whole-mount brains; some olfactory areas and the amygdala were significantly labeled at TM10.5–TM14.5 (blue arrowheads 2 in Figure 2C). The labeled amygdala nuclei overlapped with, but were distinct from, those labeled by the Neurog2^{CreER}(G2A) driver (Figure 2A). Neurons in the inferior colliculus (brown arrowheads 3 in Figures 2A, 2C, and 2D) and the cochlear nucleus (green arrowheads 4 in Figures 2B–2D) were labeled following the same spatiotemporal pattern shared

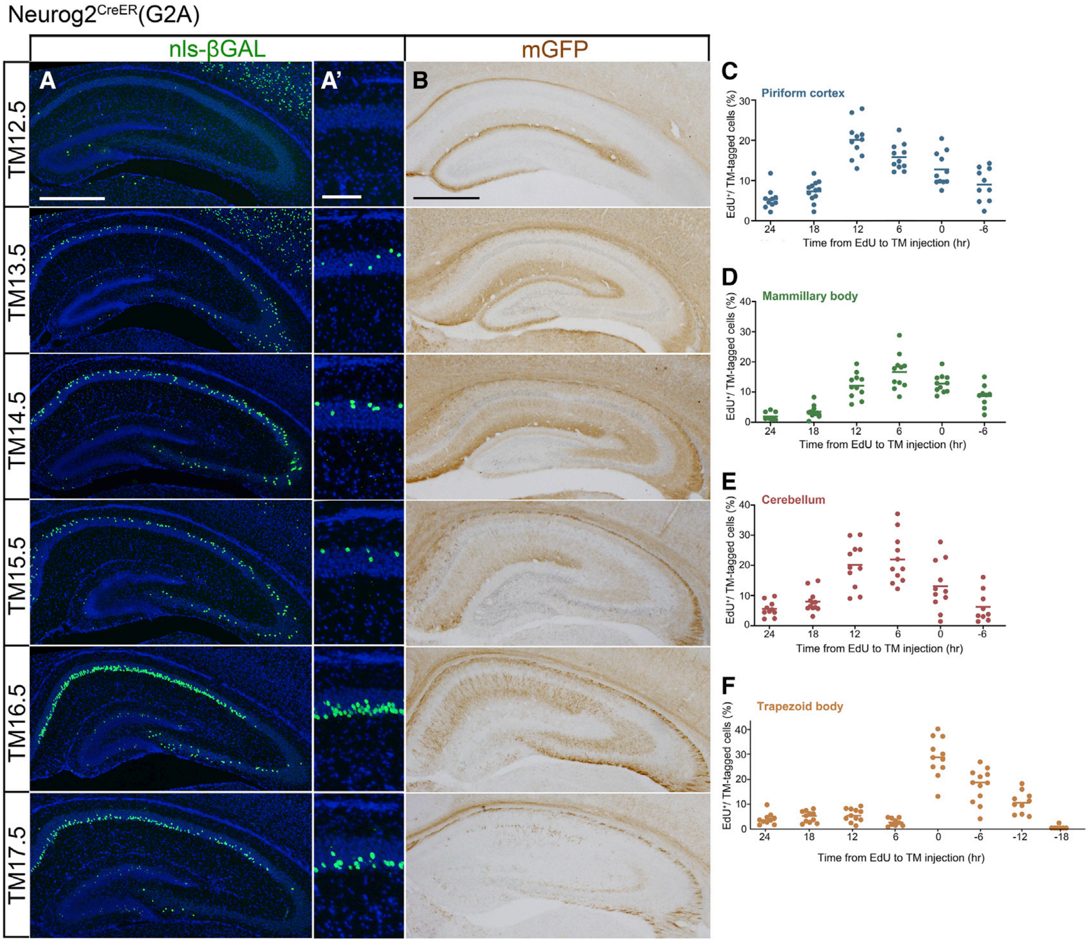

Figure 5. Hippocampus and time course of neurons tagged by Neurog2^{CreER}(G2A)

(A and B) nls-βGAL (A, A′) and mGFP (B) staining of hippocampal sections prepared from P7 mice that were tagged at different stages indicated on the left. (A′) High magnification of the CA1 pyramidal layer in (A). Neurons in the deep and superficial sublayers are labeled at TM13.5–TM15.5 and TM16.5–TM17.5, respectively. In (B), afferent axons such as the perforant path are prominently labeled at early TM12.5–TM13.5, whereas dendrites and efferent axons of hippocampal pyramidal neurons are labeled at late TM16.5–TM17.5. Scale bars, 500 μm (A and B) and 100 μm (A′).

(C–F) The proportion of EdU and nls-βGAL double-positive neurons in the piriform cortex layer II (C), mammillary body (D), midline compartment of the cerebellum (E), and the trapezoid body (F). TM was injected at the fixed TM12.5 stage, and EdU was injected at the indicated time before the TM injection. The dots show the values for individual mice, and the horizontal lines show the means. Each value was calculated from 127–254 (C), 86–395 (D), 101–331 (E) and 113–366 (F) nls-βGAL-positive neurons.

by the other drivers (Table 1). The late labeling of the cochlear nucleus after TM15.5 appeared to correspond with the second wave of neurogenesis in the dorsal cochlear nucleus reported recently (Shepard et al., 2019).

In cross-sections of the neocortex, a tangential band of neurons was labeled at the border between the neocortex and white matter at TM11.5–TM12.5 (Figure 6A). These cells appeared to be surviving subplate neurons in layer VIb (Friedlander and Torres-Reveron, 2009). The mGFP reporter selectively visualized their characteristic dense basal dendrites at the bottom of the cortical plate and widespread axons in layer 1 (Figure 6B; Clancy and Cauller, 1999). Tagging at TM13.5–TM14.5 labeled only a few cortical neurons scattered in layers VI and V (Figure 6A). These neurons were mainly positioned in the medial cortex (Figures 6C and 6D), and their axons were observed to selectively project to the medial part of the striatum (Figures 6C and 6D), exhibiting a unique feature reported for neurons in the medial prefrontal area (Gerfen et al., 2013; Mailly et al., 2013). Later-stage TM injections did not label neurons in the upper layers (Figure 6A), showing a clear contrast to the inside-out pattern throughout the cortical plate induced by the Neurog2^{CreER}(G2A) driver (Figure 4A).

Neurod4^{CreER}(D4A) driver using the *neurod4* enhancer

The Neurod4^{CreER}(D4A) driver abundantly tagged neurons in the caudal parts of the brain such as the medulla and the spinal cord (magenta arrowheads 1 in Figure 2D). The spatial pattern and time course of labeling in these caudal regions resembled those of Neurog2^{CreER}(G2A) (Figure 2A and Table 1), consistent with the idea that *neurod4* is a downstream effector of *neurog2* (Seo et al., 2007; Masserdotti et al., 2015). However, there

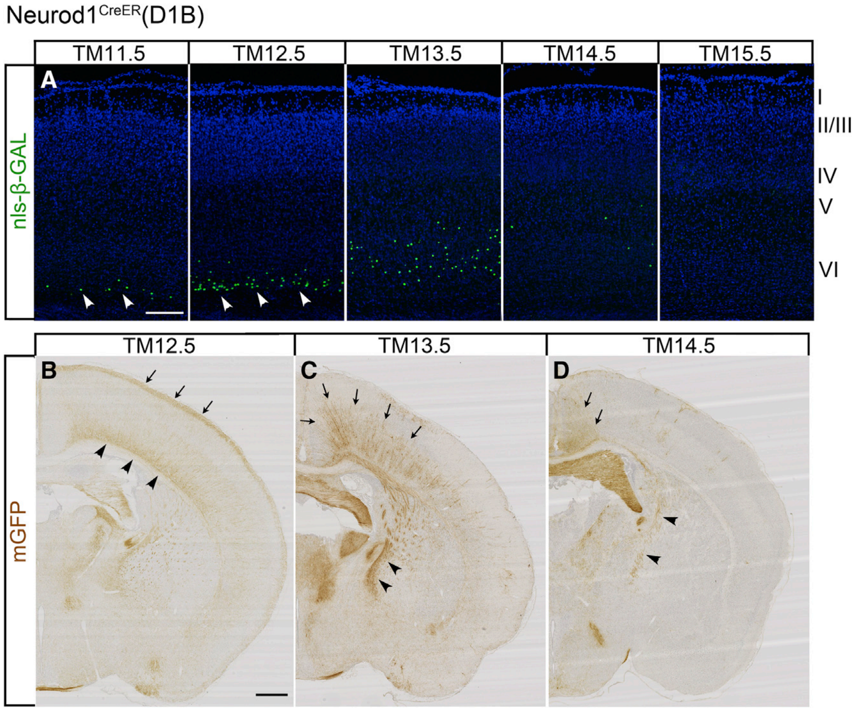

Figure 6. Cortex and telencephalic hemispheres tagged by Neurod1^{CreER}(D1B)

(A) P7 cortical sections stained for nls-βGAL. Subplate neurons in the sublayer VIb are labeled at TM11.5–TM12.5 (arrowheads). Later TM injections label only a small number of neurons in the layers V and VI. Scale bar, 200 μm.

(B–D) mGFP staining of telencephalic sections prepared from P7 mice tagged at TM12.5 (B), TM13.5 (C), and TM14.5 (D). In (B), the labeling at the bottom (arrowheads) and the top (arrows) of the cortical plate corresponds to the typical positions of basal dendrites and axons of surviving subplate neurons. During later TM stages (C and D), the tagged neurons are concentrated on the medial cortical areas (arrows), and their axons selectively project to the medial part of the striatum (arrowheads). Scale bar, 500 μm.

were differences between the two drivers; for example, the neocortex and cerebellar Purkinje cells were not significantly labeled by the Neurod4^{CreER}(D4A) driver (Figure 2D and Table 1).

This driver notably visualized long projecting axons in several brain regions. For example, mGFP-labeled optic axons from the retinal ganglion cells tagged at TM13.5–TM14.5 were targeted at the superficial layers of the superior colliculus (Figure S2D). The habenular neurons in the thalamus were generated in a lateral-to-medial gradient (Figure 7A) (Angevine, 1970; Aizawa et al., 2007), whereas their axons exhibited a periphery-to-center arrangement within the fasciculus retroflexus, which is the axon tract formed by habenular neurons (Figure 7B).

Using this driver, we also characterized the cell-cycle timing of neurons that underwent TM-induced recombination. TM injection at the fixed E14.5 stage labeled neurons in the hippocampal CA3 region (Figure 7C), habenula (Figure 7D), supramammillary nucleus (Figure 7E), and inferior colliculus (Figure 7F). The time-spaced injection of EdU indicated that neurons were most susceptible to TM-induced recombination at 6–12 h after the final DNA synthesis. Although the time course of double labeling seemed slightly delayed compared with that of Neurog2^{CreER}(G2A) (Figures 5C–5F), such a comparison might not be meaningful, as different neurons and TM stages were involved and we could not quantitatively determine the time lag in the gene expression cascade from *neurog2* to *neurod4*.

DISCUSSION

Neurogenic tagging resource

In the past few decades, various important discoveries have been made through neuronal birthdating (Bayer and Altman, 1987; Govindan et al., 2018). The neurogenic tagging resource presented in this study resolves some of the limitations of the previous method and forges a link between this traditional histological classification and the new experimental approaches and applications. Specifically, neurogenic tagging can be easily combined with various molecular genetic tools such as optogenetics and chemogenetics (Deisseroth et al., 2006; Alexander et al., 2009), which enable the functional manipulation of birthdate-classified neuronal subsets. We hope that the unique ideas of researchers will lead to important discoveries using this resource, given that the neuronal birthdate is so fundamental to organizing the neural circuitry.

This neurogenic tagging method uses the expression timing of neuronal differentiation genes; therefore, the underlying biological principle is significantly different from that of the traditional method using nucleotide analogs. The four neuronal differentiation genes exploited in our resources were selected on the basis of their seemingly transient expression during the maturation phase of neurons as shown in a previous study (Mattar et al., 2008) and databases (Allen Developmental Mouse Brain Atlas, https://developingmouse.brain-map.org; GENSAT: Gene Expression Nervous System Atlas, http://www.gensat.org/index.html). As expected, the neurons underwent *loxP* recombination 6–12 h after the final DNA synthesis in different parts of the nervous system (Figures 5C–5E and 7C–7F), and recapitulated the patterns consistent with those seen in birthdating analyses using nucleotide analogs (Table 1). In some exceptional cases, such as the trapezoid body (Figure 5F), TM-induced recombination seemed to occur within a short time window during the progenitor stage before the final DNA synthesis. In the pontine nucleus and the dorsal root ganglion (Table 1), the earlier tagging stages, compared with the previously determined birthdates, also suggest that TM-induced recombination occurs pre-mitotically in these regions. Thus, neurogenic tagging using neuronal differentiation genes appears to mark the timing of neuron commitment rather than cell-cycle exit (Hirata et al., 2019). Recently, increasing evidence has indicated that neuron commitment and cell-cycle exit are dissociable processes

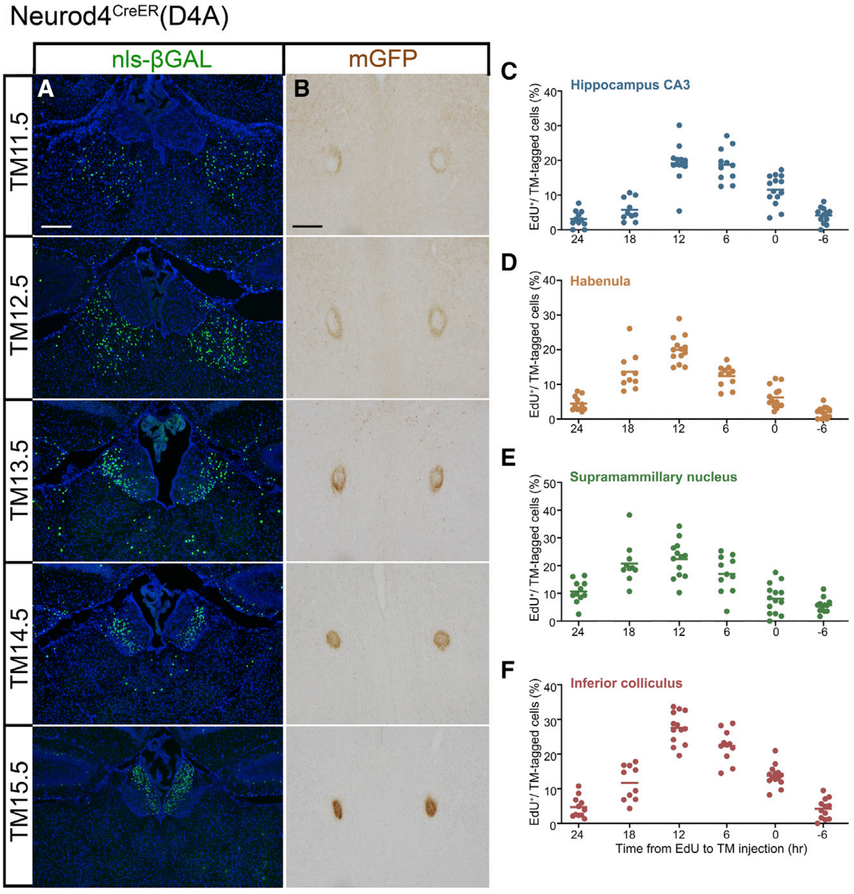

Figure 7. Habenula and time course of neurons tagged by Neurod4^{CreER}(D4A)

(A and B) nls-βGAL staining of the habenula nucleus (A) and mGFP staining of the fasciculus retroflexus (B) obtained from P7 mice that were tagged at different stages indicated on the left. Depending on the TM stage, habenular neurons are labeled from lateral to medial (A). Their axons are labeled from the periphery to the center of the fasciculus retroflexus (B). Scale bar, 200 μm.

(C–F) The proportion of EdU and nls-βGAL double-positive neurons in the hippocampus CA3 (C), habenula (D), supramammillary nucleus (E), and inferior colliculus (F). TM was injected at the fixed E14.5 stage, and EdU was injected at the indicated time before TM injection. The dots show the values of individual mice, and the horizontal lines show the means. Each value was calculated from 20–153 (C), 71–257 (D), 43–173 (E), and 52–199 (F) nls-βGAL-positive neurons.

(Imayoshi et al., 2013; Hardwick and Philpott, 2014; Oberst et al., 2019). A prime example is the cortical basal progenitor, which is the fate-restricted neural progenitor that undergoes additional cell divisions but only generates neurons (Lodato and Arlotta, 2015; Hevner, 2019). Our ongoing study shows that these cortical basal progenitors are indeed tagged by one of the neurogenic tagging driver lines.

During development, the *neurod1* gene is widely expressed in many nervous systems (Miyata et al., 1999; Mattar et al., 2008), and a short enhancer element of this gene is commonly used to study neuronal differentiation *in vivo* and *in vitro* (Guerrier et al., 2009). Somewhat contradictory to the general use of this gene, the Neurod1^{CreER}(D1B) driver tagged only a restricted subset of neurons (Figures 2C and 6). This might be because we obtained only one mouse line that exhibited significant CreER activity after the intensive production of transgenic mice by using the *neurod1* gene. Regardless, the unique characteristics of neuronal tagging by the Neurod1^{CreER}(D1B) driver shown in this study and NeuroGT demonstrate the usefulness of this driver line for some specific purposes.

The present collection of four neurogenic tagging drivers does not fully cover all brain regions. For example, the majority of neurons in the striatum and hypothalamus were unlabeled by any of the drivers. To complement the current collection a promising candidate is the *Ascl1* gene, which is expressed in the basal plate of the neural tube in a manner complementary to that of neuronal differentiation genes used in this study (Ma et al., 1997; Wilkinson et al., 2013). Furthermore, the mice that express CreER under the *Ascl1* gene enhancer have been reported to apparently show birthdate-dependent recombination (Tg(Ascl1-cre/Esr1*)1Jejo, MGI: 3767428, Battiste et al., 2007; Ascl1$^{tm1.1(cre/ERT2)Jejo}$, MGI: 4452601, Kim et al., 2008; Sudarov et al., 2011). In the future, we hope to include detailed image data of neurons tagged by Ascl1^{CreER} mice in the NeuroGT database.

To date, multiple CreER-expressing mice under the control of neuronal differentiation genes have been generated by several groups (Neurog2$^{tm1(cre/Esr1*)And}$, MGI: 2652037, Zirlinger et al., 2002; Tg(Neurog1-cre/ERT2)1Good/J, MGI: 3807088, Koundakjian et al., 2007; *Neurog2iCreERT2*, Winpenny et al., 2011; Neurog2$^{tm1(icre/ERT2*)Ggc}$, MGI: 5431772, Florio et al., 2012; Tg(Neurod1-cre/ERT2)M1Fcal, MGI: 5582823, Aprea et al., 2014). Although these mice have been primarily used for cell lineage analyses related to the manipulated genes, they are theoretically applicable to birthdate-based classification and manipulation of neurons. Our Nerog2^{CreER}(G2A) driver mouse generated by BAC transgenesis has a greater CreER activity than that under the endogenous locus in Neurog2-CreER knockin mice (Zirlinger et al., 2002). However, suitable drivers differ depending on the research purpose. It is important to note that information in the NeuroGT database can be useful even for developing a research design that does not use our mouse lines.

Features highlighted by the present neurogenic tagging analyses

Our original motivation for developing the neurogenic tagging method was to visualize axon projections of birthdate-classified

neuron subsets in the olfactory bulb in adulthood. This method was indeed effective in revealing the birthdate-dependent olfactory axon trajectories (Hirata et al., 2019). It has also been applied to examine birthdate-dependent compartments in the cerebellum (Tran-Anh et al., 2020; Zhang et al., 2020). The present analyses using the multiple neurogenic tagging drivers added interesting findings, three of which are discussed below.

Corpus callosum

Within the corpus callosum, early-born (TM12.5–TM13.5) and late-born (TM16.5–TM17.5) cortical neurons formed segregated axon bundles (Figures 4B and S1D–S1G), consistent with the current knowledge that the callosal projections are formed by a subset of layers II/III and V neurons in the neocortex (Fame et al., 2011). The segregated construction of the callosum by pioneering and following axons has been proposed (Koester and O'Leary, 1994; Rash and Richards, 2001). Although the previous studies have considered the time lag of axon arrivals from the medial and lateral cortical areas (Piper et al., 2009; Nishikimi et al., 2011; Zhou et al., 2013), the present analysis indicates that the time lag between the early-born and late-born callosal neurons in different layers also underlies the organization of this axon bundle, which is the largest in placental mammals (Suarez et al., 2014).

Fasciculus retroflexus

Neurogenic tagging revealed a periphery-to-center axon topology within the fasciculus retroflexus (Figures 7A and 7B). A previous axon-tracing analysis reported that the medial and lateral habenula nuclei project their axons into the core and shell of this tract, respectively (Herkenham and Nauta, 1979). To explain this organization, the present study provides a hint: late-growing axons might penetrate the center of the bundle pre-formed by early-growing axons. In general, for axon tracts positioned on the brain surface, late-growing axons are sequentially added to the pial superficial space, displacing the pre-existing axons deeper (Walsh et al., 1983; Walsh and Guillery, 1985; Inaki et al., 2004; Yamatani et al., 2004). Although little is known about the development of internally located axon tracts, new axons are found to grow in the center of pre-formed axon bundle in the mushroom body of *Drosophila* (Kurusu et al., 2002). Within the optic nerve, which is an isolated axon bundle, newly growing axons are positioned near the center (Walsh, 1986).

Hippocampus pyramidal neurons

Although hippocampal neurons in the single pyramidal layer have been regarded as a homogeneous population, recent studies indicate that hippocampal pyramidal neurons are in fact heterogeneous along the radial axis (Slomianka et al., 2011; Soltesz and Losonczy, 2018). The deep and superficial subsets in the layer express different genes, make different connections, and are expected to have distinct functions in different behavioral contexts (Mizuseki et al., 2011; Lee et al., 2014; Danielson et al., 2016). These subsets are assumed to differentiate at different times in an inside-out pattern (Slomianka et al., 2011; Soltesz and Losonczy, 2018). The present neurogenic tagging analysis confirmed this assumption and allowed effective classification of these neuronal subsets (Figures 5A and 5A′). An advantage of this neurogenic tagging is that this classification can now be connected to functional tests. We hope that the neurogenic tagging resource will benefit ingenious ideas that will lead to future scientific discoveries.

Limitations of the study

Neurogenic tagging is different from nucleotide-based neuronal birthdating. The timing of recombination in the cell cycle differs among neurons and appears to take place prior to the final mitosis in some neurons. It is important to consider this difference when the neurogenic tagging approach is taken, as this difference might be beneficial in some cases. In the NeuroGT database, the anatomical ontology of tagged brain regions is not yet available but will be included in the future to enable searching by anatomical regions.

STAR★METHODS

Detailed methods are provided in the online version of this paper and include the following:

- KEY RESOURCES TABLE
- RESOURCE AVAILABILITY
  - Lead contact
  - Materials availability
  - Data and code availability
- EXPERIMENTAL MODEL AND SUBJECT DETAILS
  - Mice
- METHODS DETAILS
  - Generation of neurogenic tagging mouse lines
  - Histochemistry
  - Image acquisition
  - EdU incorporation assay
  - Construction of the NeuroGT database
- QUANTIFICATION AND STATISTICAL ANALYSIS
- ADDITIONAL RESOURCES

SUPPLEMENTAL INFORMATION

Supplemental information can be found online at https://doi.org/10.1016/j.crmeth.2021.100012.

ACKNOWLEDGMENTS

The authors thank Yash Hiromi and Yan Zhu for their comments on the manuscript, Yumiko Hatanaka and Hidenori Aizawa for providing helpful information, and Pierre Chambon for *CreER*^{T2}, Junji Takeda for p23loxZeo and p24loxZeo, and Silvia Arber for Tau$^{mGFP\text{-}nLacZ}$ mice. This research was supported by MEXT/JSPS KAKENHI grants (19HP7002 and 20H03345) to T.H., Platform for Advanced Bioimaging Support (JP16H06280) and ROIS Challenging Exploratory Research Projects for the Future Grant awarded to T.H., and JSPS KAKENHI grant (JP18H05412) to S.O.

AUTHOR CONTRIBUTIONS

T.H. conceived the research, performed the experiments, analyzed the data, and wrote the manuscript with help from other authors. G.S. and H.K. established the mouse lines. S.O. and T.F. converted the images of neurogenic-tagged serial sections into high-resolution digital data. Y.T., H.I., and S.O. processed the image data and constructed the NeuroGT database.

DECLARATION OF INTERESTS

The authors declare no competing interests.

Received: January 11, 2021
Revised: April 2, 2021
Accepted: April 23, 2021
Published: May 21, 2021

REFERENCES

Aizawa, H., Goto, M., Sato, T., and Okamoto, H. (2007). Temporally regulated asymmetric neurogenesis causes left-right difference in the zebrafish habenular structures. Dev. Cell *12*, 87–98.

Alexander, G.M., Rogan, S.C., Abbas, A.I., Armbruster, B.N., Pei, Y., Allen, J.A., Nonneman, R.J., Hartmann, J., Moy, S.S., Nicolelis, M.A., et al. (2009). Remote control of neuronal activity in transgenic mice expressing evolved G protein-coupled receptors. Neuron *63*, 27–39.

Altman, J., and Bayer, S.A. (1981). Time of origin of neurons of the rat inferior colliculus and the relations between cytogenesis and tonotopic order in the auditory pathway. Exp. Brain Res. *42*, 411–423.

Angevine, J.B., Jr. (1970). Time of neuron origin in the diencephalon of the mouse. An autoradiographic study. J. Comp. Neurol. *139*, 129–187.

Aprea, J., Nonaka-Kinoshita, M., and Calegari, F. (2014). Generation and characterization of Neurod1-CreER(T2) mouse lines for the study of embryonic and adult neurogenesis. Genesis *52*, 870–878.

Battiste, J., Helms, A.W., Kim, E.J., Savage, T.K., Lagace, D.C., Mandyam, C.D., Eisch, A.J., Miyoshi, G., and Johnson, J.E. (2007). Ascl1 defines sequentially generated lineage-restricted neuronal and oligodendrocyte precursor cells in the spinal cord. Development *134*, 285–293.

Bayer, S.A., and Altman, J. (1987). Directions in neurogenetic gradients and patterns of anatomical connections in the telencephalon. Prog. Neurobiol. *29*, 57–106.

Bayer, S.A., and Altman, J. (1991). Neocortical Development (Raven Press).

Caviness, V.S., Jr. (1973). Time of neuron origin in the hippocampus and dentate gyrus of normal and reeler mutant mice: an autoradiographic analysis. J. Comp. Neurol. *151*, 113–120.

Caviness, V.S., Jr. (1982). Neocortical histogenesis in normal and reeler mice: a developmental study based upon [^{3}H]thymidine autoradiography. Brain Res. *256*, 293–302.

Clancy, B., and Cauller, L.J. (1999). Widespread projections from subgriseal neurons (layer VII) to layer I in adult rat cortex. J. Comp. Neurol. *407*, 275–286.

Danielson, N.B., Zaremba, J.D., Kaifosh, P., Bowler, J., Ladow, M., and Losonczy, A. (2016). Sublayer-specific coding dynamics during spatial navigation and learning in hippocampal area CA1. Neuron *91*, 652–665.

Deisseroth, K., Feng, G., Majewska, A.K., Miesenbock, G., Ting, A., and Schnitzer, M.J. (2006). Next-generation optical technologies for illuminating genetically targeted brain circuits. J. Neurosci. *26*, 10380–10386.

Edwards, M.A., Caviness, V.S., Jr., and Schneider, G.E. (1986). Development of cell and fiber lamination in the mouse superior colliculus. J. Comp. Neurol. *248*, 395–409.

Fame, R.M., MacDonald, J.L., and Macklis, J.D. (2011). Development, specification, and diversity of callosal projection neurons. Trends Neurosci. *34*, 41–50.

Feil, R., Wagner, J., Metzger, D., and Chambon, P. (1997). Regulation of Cre recombinase activity by mutated estrogen receptor ligand-binding domains. Biochem. Biophys. Res. Commun. *237*, 752–757.

Florio, M., Leto, K., Muzio, L., Tinterri, A., Badaloni, A., Croci, L., Zordan, P., Barili, V., Albieri, I., Guillemot, F., et al. (2012). Neurogenin 2 regulates progenitor cell-cycle progression and Purkinje cell dendritogenesis in cerebellar development. Development *139*, 2308–2320.

Forster, E., Zhao, S., and Frotscher, M. (2006). Laminating the hippocampus. Nat. Rev. Neurosci. *7*, 259–267.

Friedlander, M.J., and Torres-Reveron, J. (2009). The changing roles of neurons in the cortical subplate. Front Neuroanat. *3*, 15.

Gerfen, C.R., Paletzki, R., and Heintz, N. (2013). GENSAT BAC cre-recombinase driver lines to study the functional organization of cerebral cortical and basal ganglia circuits. Neuron *80*, 1368–1383.

Govindan, S., Oberst, P., and Jabaudon, D. (2018). In vivo pulse labeling of isochronic cohorts of cells in the central nervous system using FlashTag. Nat. Protoc. *13*, 2297–2311.

Guerrier, S., Coutinho-Budd, J., Sassa, T., Gresset, A., Jordan, N.V., Chen, K., Jin, W.L., Frost, A., and Polleux, F. (2009). The F-BAR domain of srGAP2 induces membrane protrusions required for neuronal migration and morphogenesis. Cell *138*, 990–1004.

Hardwick, L.J., and Philpott, A. (2014). Nervous decision-making: to divide or differentiate. Trends Genet. *30*, 254–261.

Hashimoto, M., and Mikoshiba, K. (2003). Mediolateral compartmentalization of the cerebellum is determined on the "birth date" of Purkinje cells. J. Neurosci. *23*, 11342–11351.

Herkenham, M., and Nauta, W.J. (1979). Efferent connections of the habenular nuclei in the rat. J. Comp. Neurol. *187*, 19–47.

Hevner, R.F. (2019). Intermediate progenitors and Tbr2 in cortical development. J. Anat. *235*, 616–625.

Hinds, J.W. (1968). Autoradiographic study of histogenesis in the mouse olfactory bulb. I. Time of origin of neurons and neuroglia. J. Comp. Neurol. *134*, 287–304.

Hippenmeyer, S., Vrieseling, E., Sigrist, M., Portmann, T., Laengle, C., Ladle, D.R., and Arber, S. (2005). A developmental switch in the response of DRG neurons to ETS transcription factor signaling. PLoS Biol. *3*, e159.

Hirata, T., and Iwai, L. (2019). Timing matters: a strategy for neurons to make diverse connections. Neurosci. Res. *138*, 79–83.

Hirata, T., Shioi, G., Abe, T., Kiyonari, H., Kato, S., Kobayashi, K., Mori, K., and Kawasaki, T. (2019). A novel birthdate-labeling method reveals segregated parallel projections of mitral and external tufted cells in the main olfactory system. eNeuro *6*. https://doi.org/10.1523/ENEURO.0234-19.2019.

Hoerder-Suabedissen, A., and Molnar, Z. (2013). Molecular diversity of early-born subplate neurons. Cereb. Cortex *23*, 1473–1483.

Imayoshi, I., Isomura, A., Harima, Y., Kawaguchi, K., Kori, H., Miyachi, H., Fujiwara, T., Ishidate, F., and Kageyama, R. (2013). Oscillatory control of factors determining multipotency and fate in mouse neural progenitors. Science *342*, 1203–1208.

Inaki, K., Nishimura, S., Nakashiba, T., Itohara, S., and Yoshihara, Y. (2004). Laminar organization of the developing lateral olfactory tract revealed by differential expression of cell recognition molecules. J. Comp. Neurol. *479*, 243–256.

Kawakami, A., Kitsukawa, T., Takagi, S., and Fujisawa, H. (1996). Developmentally regulated expression of a cell surface protein, neuropilin, in the mouse nervous system. J. Neurobiol. *29*, 1–17.

Kawauchi, D., Taniguchi, H., Watanabe, H., Saito, T., and Murakami, F. (2006). Direct visualization of nucleogenesis by precerebellar neurons: involvement of ventricle-directed, radial fibre-associated migration. Development *133*, 1113–1123.

Kim, E.J., Battiste, J., Nakagawa, Y., and Johnson, J.E. (2008). Ascl1 (Mash1) lineage cells contribute to discrete cell populations in CNS architecture. Mol. Cell Neurosci. *38*, 595–606.

Kim, E.J., Hori, K., Wyckoff, A., Dickel, L.K., Koundakjian, E.J., Goodrich, L.V., and Johnson, J.E. (2011). Spatiotemporal fate map of neurogenin1 (Neurog1) lineages in the mouse central nervous system. J. Comp. Neurol. *519*, 1355–1370.

Koester, S.E., and O'Leary, D.D. (1994). Axons of early generated neurons in cingulate cortex pioneer the corpus callosum. J. Neurosci. *14*, 6608–6620.

Koundakjian, E.J., Appler, J.L., and Goodrich, L.V. (2007). Auditory neurons make stereotyped wiring decisions before maturation of their targets. J. Neurosci. *27*, 14078–14088.

CellPress
OPEN ACCESS

Kurusu, M., Awasaki, T., Masuda-Nakagawa, L.M., Kawauchi, H., Ito, K., and Furukubo-Tokunaga, K. (2002). Embryonic and larval development of the Drosophila mushroom bodies: concentric layer subdivisions and the role of fasciclin II. Development *129*, 409–419.

Lawson, S.N., and Biscoe, T.J. (1979). Development of mouse dorsal root ganglia: an autoradiographic and quantitative study. J. Neurocytol. *8*, 265–274.

Lee, S.H., Marchionni, I., Bezaire, M., Varga, C., Danielson, N., Lovett-Barron, M., Losonczy, A., and Soltesz, I. (2014). Parvalbumin-positive basket cells differentiate among hippocampal pyramidal cells. Neuron *82*, 1129–1144.

Lodato, S., and Arlotta, P. (2015). Generating neuronal diversity in the mammalian cerebral cortex. Annu. Rev. Cell Dev. Biol. *31*, 699–720.

Ma, Q., Sommer, L., Cserjesi, P., and Anderson, D.J. (1997). Mash1 and neurogenin1 expression patterns define complementary domains of neuroepithelium in the developing CNS and are correlated with regions expressing notch ligands. J. Neurosci. *17*, 3644–3652.

Mailly, P., Aliane, V., Groenewegen, H.J., Haber, S.N., and Deniau, J.M. (2013). The rat prefrontostriatal system analyzed in 3D: evidence for multiple interacting functional units. J. Neurosci. *33*, 5718–5727.

Masserdotti, G., Gillotin, S., Sutor, B., Drechsel, D., Irmler, M., Jorgensen, H.F., Sass, S., Theis, F.J., Beckers, J., Berninger, B., et al. (2015). Transcriptional mechanisms of proneural factors and REST in regulating neuronal reprogramming of astrocytes. Cell Stem Cell *17*, 74–88.

Mattar, P., Langevin, L.M., Markham, K., Klenin, N., Shivji, S., Zinyk, D., and Schuurmans, C. (2008). Basic helix-loop-helix transcription factors cooperate to specify a cortical projection neuron identity. Mol. Cell Biol. *28*, 1456–1469.

McConnell, J., and Angevine, J.B., Jr. (1983). Time of neuron origin in the amygdaloid complex of the mouse. Brain Res. *272*, 150–156.

McConnell, S.K. (1989). The determination of neuronal fate in the cerebral cortex. Trends Neurosci. *12*, 342–349.

Miyata, T., Maeda, T., and Lee, J.E. (1999). NeuroD is required for differentiation of the granule cells in the cerebellum and hippocampus. Genes Dev. *13*, 1647–1652.

Mizuseki, K., Diba, K., Pastalkova, E., and Buzsaki, G. (2011). Hippocampal CA1 pyramidal cells form functionally distinct sublayers. Nat. Neurosci. *14*, 1174–1181.

Namba, K., Sugihara, I., and Hashimoto, M. (2011). Close correlation between the birth date of Purkinje cells and the longitudinal compartmentalization of the mouse adult cerebellum. J. Comp. Neurol. *519*, 2594–2614.

Nishikimi, M., Oishi, K., Tabata, H., Torii, K., and Nakajima, K. (2011). Segregation and pathfinding of callosal axons through EphA3 signaling. J. Neurosci. *31*, 16251–16260.

Nornes, H.O., and Carry, M. (1978). Neurogenesis in spinal cord of mouse: an autoradiographic analysis. Brain Res. *159*, 1–6.

Oberst, P., Fievre, S., Baumann, N., Concetti, C., Bartolini, G., and Jabaudon, D. (2019). Temporal plasticity of apical progenitors in the developing mouse neocortex. Nature *573*, 370–374.

Pierce, E.T. (1973). Time of origin of neurons in the brain stem of the mouse. Prog. Brain Res. *40*, 53–65.

Piper, M., Plachez, C., Zalucki, O., Fothergill, T., Goudreau, G., Erzurumlu, R., Gu, C., and Richards, L.J. (2009). Neuropilin 1-Sema signaling regulates crossing of cingulate pioneering axons during development of the corpus callosum. Cereb. Cortex *19* (*Suppl 1*), i11–21.

Rash, B.G., and Richards, L.J. (2001). A role for cingulate pioneering axons in the development of the corpus callosum. J. Comp. Neurol. *434*, 147–157.

Saga, Y., Yagi, T., Ikawa, Y., Sakakura, T., and Aizawa, S. (1992). Mice develop normally without tenascin. Genes Dev. *6*, 1821–1831.

Sarma, A.A., Richard, M.B., and Greer, C.A. (2011). Developmental dynamics of piriform cortex. Cereb. Cortex *21*, 1231–1245.

Sato, Y., Hirata, T., Ogawa, M., and Fujisawa, H. (1998). Requirement for early-generated neurons recognized by monoclonal antibody lot1 in the formation of lateral olfactory tract. J. Neurosci. *18*, 7800–7810.

Schindelin, J., Arganda-Carreras, I., Frise, E., Kaynig, V., Longair, M., Pietzsch, T., Preibisch, S., Rueden, C., Saalfeld, S., Schmid, B., et al. (2012). Fiji: an open-source platform for biological-image analysis. Nat. Methods *9*, 676–682.

Seo, S., Lim, J.W., Yellajoshyula, D., Chang, L.W., and Kroll, K.L. (2007). Neurogenin and NeuroD direct transcriptional targets and their regulatory enhancers. EMBO J. *26*, 5093–5108.

Shepard, A.R., Scheffel, J.L., and Yu, W.M. (2019). Relationships between neuronal birthdates and tonotopic positions in the mouse cochlear nucleus. J. Comp. Neurol. *527*, 999–1011.

Shimada, M., and Nakamura, T. (1973). Time of neuron origin in mouse hypothalamic nuclei. Exp. Neurol. *41*, 163–173.

Slomianka, L., Amrein, I., Knuesel, I., Sorensen, J.C., and Wolfer, D.P. (2011). Hippocampal pyramidal cells: the reemergence of cortical lamination. Brain Struct. Funct. *216*, 301–317.

Soltesz, I., and Losonczy, A. (2018). CA1 pyramidal cell diversity enabling parallel information processing in the hippocampus. Nat. Neurosci. *21*, 484–493.

Soriano, P. (1999). Generalized lacZ expression with the ROSA26 Cre reporter strain. Nat. Genet. *21*, 70–71.

Suarez, R., Gobius, I., and Richards, L.J. (2014). Evolution and development of interhemispheric connections in the vertebrate forebrain. Front. Hum. Neurosci. *8*, 497.

Sudarov, A., Turnbull, R.K., Kim, E.J., Lebel-Potter, M., Guillemot, F., and Joyner, A.L. (2011). Ascl1 genetics reveals insights into cerebellum local circuit assembly. J. Neurosci. *31*, 11055–11069.

Suzuki, I.K., and Hirata, T. (2013). Neocortical neurogenesis is not really "neo": a new evolutionary model derived from a comparative study of chick pallial development. Dev. Growth Differ. *55*, 173–187.

Szabo, N.E., Haddad-Tovolli, R., Zhou, X., and Alvarez-Bolado, G. (2015). Cadherins mediate sequential roles through a hierarchy of mechanisms in the developing mammillary body. Front. Neuroanat. *9*, 29.

Tohsato, Y., Ho, K.H.L., Kyoda, K., and Onami, S. (2016). SSBD: a database of quantitative data of spatiotemporal dynamics of biological phenomena. Bioinformatics *32*, 3471–3479.

Toma, K., Kumamoto, T., and Hanashima, C. (2014). The timing of upper-layer neurogenesis is conferred by sequential derepression and negative feedback from deep-layer neurons. J. Neurosci. *34*, 13259–13276.

Tran-Anh, K., Zhang, J., Nguyen-Minh, V.T., Fujita, H., Hirata, T., and Sugihara, I. (2020). Common origin of the cerebellar dual somatotopic areas revealed by tracking embryonic Purkinje cell clusters with birthdate tagging. eNeuro *7*. https://doi.org/10.1523/ENEURO.0251-20.2020.

Walsh, C. (1986). Age-related fiber order in the ferret's optic nerve and optic chiasm. J. Neurosci. *6*, 1635–1642.

Walsh, C., and Guillery, R.W. (1985). Age-related fiber order in the optic tract of the ferret. J. Neurosci. *5*, 3061–3069.

Walsh, C., Polley, E.H., Hickey, T.L., and Guillery, R.W. (1983). Generation of cat retinal ganglion cells in relation to central pathways. Nature *302*, 611–614.

Wilkinson, G., Dennis, D., and Schuurmans, C. (2013). Proneural genes in neocortical development. Neuroscience *253*, 256–273.

Winpenny, E., Lebel-Potter, M., Fernandez, M.E., Brill, M.S., Gotz, M., Guillemot, F., and Raineteau, O. (2011). Sequential generation of olfactory bulb glutamatergic neurons by Neurog2-expressing precursor cells. Neural Dev. *6*, 12.

Yamatani, H., Sato, Y., Fujisawa, H., and Hirata, T. (2004). Chronotopic organization of olfactory bulb axons in the lateral olfactory tract. J. Comp. Neurol. *475*, 247–260.

Zeng, S.J., Lin, Y.T., Tian, C.P., Song, K.J., Zhang, X.W., and Zuo, M.X. (2009). Evolutionary significance of delayed neurogenesis in the core

versus shell auditory areas of *Mus musculus*. J. Comp. Neurol. *515*, 600–613.

Zhang, J., Tran-Anh, K., Hirata, T., and Sugihara, I. (2020). Striped distribution pattern of Purkinje cells of different birthdates in the mouse cerebellar cortex studied with the Neurog2-CreER transgenic line. Neuroscience *462*, 122–140.

Zhou, J., Wen, Y., She, L., Sui, Y.N., Liu, L., Richards, L.J., and Poo, M.M. (2013). Axon position within the corpus callosum determines contralateral cortical projection. Proc. Natl. Acad. Sci. U S A *110*, E2714–E2723.

Zirlinger, M., Lo, L., McMahon, J., McMahon, A.P., and Anderson, D.J. (2002). Transient expression of the bHLH factor neurogenin-2 marks a subpopulation of neural crest cells biased for a sensory but not a neuronal fate. Proc. Natl. Acad. Sci. U S A *99*, 8084–8089.

STAR★METHODS

KEY RESOURCES TABLE

REAGENT or RESOURCE	SOURCE	IDENTIFIER
**Antibodies**		
Chicken anti-β-GAL	Abcam	Cat# ab9361; RRID: AB_307210
Rabbit anti-GFP	MBL International	Cat# 598; RRID: AB_591816
Chicken anti-GFP antibody	Abcam	Cat# ab13970; RRID: AB_300798
Rat monoclonal anti-CTIP2 antibody	Abcam	Cat# ab51502; RRID: AB_882455
Mouse monoclonal anti-SATB2 antibody	Abcam	Cat# ab51502; RRID: AB_882455
Rabbit anti-GABA antibody	Sigma-Aldrich	Cat# A2052; RRID: AB_477652
Rabbit anti-NRP1 antibody	Kawakami et al., 1996	
Donkey biotin-SP-conjugated anti-rabbit IgG	Jackson Immunoresearch	Cat# 711-065-152; RRID:AB_2340593
Donkey Alexa488-conjugated anti-rabbit IgG	Life Technologies	Cat# A-21206; RRID: AB_141708
Donkey Cy3-conjugated anti-rabbit IgG	Jackson Immunoresearch	Cat# 711-165-152; RRID: AB_2307443
Donkey Alexa488-conjugated anti-chicken IgY	Jackson Immunoresearch	Cat# 703-545-155; RRID: AB_2340375
Donkey biotin-SP-conjugated anti-chicken IgY	Jackson Immunoresearch	Cat# 703-065-155; RRID: AB_2313596
Donkey Cy3-conjugated anti-rat IgG	Jackson Immunoresearch	Cat# 712-166-153; RRID: AB_2340669
Donkey Cy3- conjugated anti-mouse IgG	Jackson Immunoresearch	Cat# 715-165-150; RRID: AB_2340813
**Chemicals, peptides, and recombinant proteins**		
Tamoxifen	Sigma-Aldrich	T5648; CAS: 10540-29-1
Corn oil	Sigma-Aldrich	C8267; CAS: 8001-30-7
Progesterone	Fujifilm Wako	161-14531; CAS: 57-83-0
5-ethynyl-2’-deoxyuridine (EdU)	Tokyo Chemical Industry	E1057; CAS: 61135-33-9
Alexa555 azide triethylammonium salt	Thermo Fisher Scientific	A20012
X-gal	Fujifilm Wako	029-15043; CAS: 7240-90-6
Elite ABC kit	Vectastain	PK-6100;<br>RRID: AB_2336819
DAB	Dojindo	347-00904; CAS: 7411-49-5
**Deposited data**		
NeuroGT database	This paper	https://ssbd.riken.jp/neurogt/
Raw images for NeuroGT database and all figures, and counts of EdU-positive and -negative neurons	This paper	SSBD:repository: https://doi.org/10.24631/ssbd.repos.2021.03.001
**Experimental models: organisms/strains**		
Mouse: C57BL/6	Japan SLC	RRID: MGI:5295404
Mouse: ICR	Japan SLC	RRID: MGI:5462094
Mouse: Neurog2^{CreER}(G2A);<br>C57BL/6-Tg(Neurog2-cre/ERT2)G2ATahi	Hirata et al., 2019	Accession # CDB0512T−1: http://www2.clst.riken.jp/arg/TG%20mutant%20mice%20list.html
Mouse: Neurog1^{CreER}(G1C);<br>C57BL/6-Tg(Neurog1-cre/ERT2)G1CTahi	This paper	Accession # CDB0526T: http://www2.clst.riken.jp/arg/TG%20mutant%20mice%20list.html
Mouse: Neurod1^{CreER}(D1B);<br>C57BL/6-Tg(Neurod1-cre/ERT2)D1BTahi	This paper	Accession #: CDB0510T: http://www2.clst.riken.jp/arg/TG%20mutant%20mice%20list.html

(*Continued on next page*)

Continued		
REAGENT or RESOURCE	SOURCE	IDENTIFIER
Mouse: Neurod4^{CreER}(D4A); C57BL/6-Tg(Neurod4-cre/ERT2) D4ATahi	This paper	Accession # CDB0511T: http://www2.clst.riken.jp/arg/TG%20mutant%20mice%20list.html
Mouse: Tau$^{mGFP-nLacZ}$; 129P2-Mapt^{tm2Arbr}	Hippenmeyer et al., 2005	RRID: IMSR_JAX:021162
Mouse: ROSA26R-Cre; Gt(ROSA)26Sor^{tm1Sor}	Soriano, 1999	MGI: 1861932
Oligonucleotides		
Cre primer1: 5'-TAAAGATATCTCACGTA CTGACGGTG-3'	Hirata et al., 2019	N/A
Cre primer2: 5'-TCTCTGACCAGAG TCATCCTTAGC-3'	Hirata et al., 2019	N/A
Recombinant DNA		
BAC clone RP24-347K19	BACPAC Resource Center	RP24-347K19
BAC clone RP23-280C11	BACPAC Resource Center	RP23-280C11
BAC clone RP23-55O18	BACPAC Resource Center	RP23-55O18
CreER^{T2}	Feil et al., 1997	N/A
p23loxZeo	Dr. Junji Takeda, Osaka University	N/A
p24loxZeo	Dr. Junji Takeda, Osaka University	N/A
Software and algorithms		
Cell Sens Standard (ver.1.16)	Olympus	N/A
Fluoview FV-ASW (ver 4.02)	Olympus	N/A
SCN400 Client (version 2.2.0.3789)	Leica	N/A
Photoshop CC 2021 (22.2.0)	Adobe	N/A
Fiji / ImageJ (2.0.0)	Schindelin et al., 2012	RRID: SCR_002285
Microsoft Excel (Mac 2019, 16.46)	Microsoft	N/A
GraphPad Prism 8	GraphPad	N/A
Other		
Slide scanner (SCN400)	Leica	N/A

RESOURCE AVAILABILITY

Lead contact

Further information and requests for resources and reagents should be directed to Tatsumi Hirata (tathirat@nig.ac.jp).

Materials availability

The neurogenic tagging mouse lines are deposited at RIKEN BDR (http://www2.clst.riken.jp/arg/TG%20mutant%20mice%20list.html) and provided with a completed materials transfer agreement. The accession numbers are as follows: Neurog2^{CreER}(G2A) driver, CDB0512T−1; Neurog1^{CreER}(G1C) driver, CDB0526T; Neurod1^{CreER}(D1B) driver CDB0510T; Neurod4^{CreER}(D4A) driver, CDB0511T. All other reagents generated in this study are available from the Lead Contact.

Data and code availability

All images acquired in this study are available in the NeuroGT database at https://ssbd.riken.jp/neurogt/. Raw images and unprocessed data related to this study and NeuroGT are archived in SSBD:repository (https://doi.org/10.24631/ssbd.repos.2021.03.001).

EXPERIMENTAL MODEL AND SUBJECT DETAILS

Mice

C57BL/6 wild-type mice (RRID: MGI: 5295404) and outbred ICR foster mothers (RRID: MGI: 5462094) were purchased from Japan SLC Inc. The Tau$^{mGFP-nLacZ}$ reporter mice, officially known as 129P2-Mapt^{tm2Arbr} (RRID: IMSR_JAX: 021162, Hippenmeyer et al., 2005), with a CD-1 mixed background were provided by Dr. Silvia Arber at Friedrich Miescher Institute for Biomedical Research and backcrossed with C57BL/6 wild-type mice for at least four generations before use in this study. All mice were maintained in the animal facility of the National Institute of Genetics or RIKEN BDR. They were group housed in conventional cages under controlled conditions (temperature 23 ± 2°C, humidity 50 ± 10%, 12 h light-dark cycle), and food and water were provided *ad libitum*. All procedures for the care and treatment of mice were approved by the Institutional Animal Committees and carried out according to their guidelines.

Heterozygous neurogenic tagging driver mice were mated with homozygous reporter mice. The day on which a vaginal plug was detected and the day of birth were designated as embryonic day 0.5 (E0.5) and postnatal day 0 (P0), respectively. TM treatment was performed by intraperitoneally injecting a staged pregnant mouse with 250 μL of corn oil (C8267, CAS#8001-30-7, Sigma-Aldrich) containing 9 mM tamoxifen (T5648, CAS#10540-29-1, Sigma-Aldrich) and 5 mM progesterone (161-14531, CAS# 57-83-0, Fujifilm Wako). As TM often delays delivery, when pups were not born by E19.5, they were collected by caesarian delivery and given to ICR foster mothers. Brain sampling was performed indiscriminately once pups grew up to the appropriate age, and only brains of the desired genotype were later selected by PCR genotyping of the reserved tissues for CreER internal sequences. At the time of sampling, the sex of the mice was still obscure and therefore undetermined; for the Nerog2^{CreER}(G2A) driver, it is highly likely that only male mice were sampled because the transgene seems to be located on the Y chromosome (Hirata et al., 2019).

METHODS DETAILS

Generation of neurogenic tagging mouse lines

The Neurog2^{CreER}(G2A), officially named C57BL/6-Tg(Neurog2-cre/ERT2)G2ATahi, (accession No. CDB0512T−1: http://www2.clst.riken.jp/arg/TG%20mutant%20mice%20list.html) was established as described previously (Hirata et al., 2019).

The Neurog1^{CreER}(G1C) line, officially named C57BL/6-Tg(Neurog1-cre/ERT2)G1CTahi, was generated using the genomic BAC clone RP24-347K19 encoding the mouse *neurog1*, which was obtained from the BACPAC Resource Center (Children's Hospital Oakland Research Institute, Oakland, CA). The entire coding sequence of exon1 was replaced by *CreER*(*T2*) (Feil et al., 1997) (gifted by Dr. Pierre Chambon), and the *loxP* site in the vector backbone (pTARBAC) was deleted by replacement with the zeocin resistance gene in the p24loxZeo (gifted by Dr. Junji Takeda). For the Neurod1^{CreER}(D1B) line, officially named C57BL/6-Tg(Neurod1-cre/ERT2) D1BTahi, the *CreER*(*T2*) coding sequence was inserted at the first ATG sequence of exon2 in the BAC clone RP23-280C11 encoding the mouse *neurod1* (BACPAC Resource Center). For the Neurod4^{CreER}(D4A) line, officially named C57BL/6-Tg(Neurod4-cre/ERT2) D4ATahi, the *CreER*(*T2*) coding sequence was inserted at the first ATG sequence of exon2 in the BAC clone RP23-55O18 encoding the mouse *neurod4* (BACPAC Resource Center), and the *loxP* site in the vector backbone (pBACe3.6) was deleted by replacement with the zeocin resistance gene in the p23loxZeo (gifted by Dr. Junji Takeda). Except for the above-mentioned wild-type *loxP* sequences, the BAC vector backbone sequences, including a few genes, were unmodified.

The constructed BAC recombinants were injected into fertilized eggs with a C57BL/6 background, and the tail genomic DNA of the resulting mice was assayed by PCR for the integration of the transgene. Transgene containment was determined by PCR using internal Cre recombinase primers 5'-TAAAGATATCTCACGTACTGACGGTG-3' and 5'-TCTCTGACCAGAGTCATCCTTAGC-3', resulting in the amplification of 300-bp fragments. In total, eleven, two, and five mice were found to have random integrations of the transgenes for *neurog1*, *neurod1*, and *neurod4*, respectively, including multiple BAC constructs for each gene. These mouse lines were assayed for CreER activity by crossing them with ROSA26R Cre reporter mice, officially named Gt(ROSA)26Sor^{tm1Sor} (RRID: MGI: 1861932, Soriano, 1999). After crossing, pregnant mice were injected with TM solution at E12.5 or E14.5. Embryos were dissected from the dam at E18.5-E19.5, and their isolated brains were whole-mount stained with X-gal (5-bromo-4-chloro-3-indoyl-f3-D-galactopyranoside, 029-15043, CAS#7240-90-6, Fujifilm Wako) as will be described later. Through this screen, Neurog1^{CreER}(G1C), Neurod1^{CreER}(D1B), and Neurod4^{CreER}(D4A) were identified by the highest recombination rate among the transgenic mice for each gene.

Histochemistry

The mice were anesthetized and transcardially perfused with 4% paraformaldehyde (PFA)/phosphate-buffered saline (PBS). For whole-mount visualization of the nls-β-GAL signals, the brains were immediately dissected and immersed in PBS containing 1 mg/mL of X-gal, 5 mM $K_3[Fe(CN)_6]$, 5 mM $K_4[Fe(CN)_6]$, 2 mM $MgCl_2$, and 1% Tween 20 for 2.5 hours at 37°C (Saga et al., 1992). To prepare cryosections, the dissected brains were further fixed with 4% PFA/PBS for 8–24 hours, immersed in 30% sucrose in PBS, and frozen in a 2:1 mixture of OCT compound (Sakura Finetek) and 30% sucrose/PBS. Coronal sections of 20-μm thickness were cut on a cryostat, and mounted on MAS-coated glass slides (Matsunami Glass).

The sections were washed with 10 mM Tris-HCl (pH 7.4), 130 mM NaCl and 0.1% Tween 20 (TBST) and incubated with rabbit anti-GFP antibody (1:1000, #598, MBL International Cat# 598, RRID: AB_591816) diluted in PBS containing 0.1% Tween 20 and 0.5%

blocking reagent (FP1020, PerkinElmer) overnight at 4°C. After treatment with 0.7% H_2O_2 in methanol for 5 min at 4°C, antibody labeling was detected using donkey biotin-SP-conjugated anti-rabbit IgG antibody (1:1000, Jackson Immunoresearch Cat# 711-065-152. RRID: AB_2340593), amplified with the Elite ABC kit (1:300, PK-6100, Vectastain, RRID: AB_2336819), and visualized in TBST containing 0.13 mg/mL 3,3'-diaminobenzidine tetrahydrochloride (DAB, 347-00904, CAS#7411-49-5, Dojindo) and 0.03% H_2O_2. In some specimens, the binding of anti-GFP antibodies was fluorescently visualized with donkey Alexa488-conjugated anti-rabbit IgG (1:1000, Life Technologies Cat# A-21206, RRID: AB_141708). For antibody staining of nls-β-GAL, the sections were heat-treated in an antigen retrieval solution (HistoVT One, Nacalai Tesque) by autoclaving at 105°C for 2 min and incubated with chicken anti-βGAL antibody (1:2000, Abcam Cat# ab9361, RRID: AB_307210) diluted in PBS containing 0.1% Tween 20 and 0.5% blocking reagent overnight at 4°C. The bound antibodies were detected with donkey Alexa488-conjugated anti-chicken IgY (1:1000, Jackson Immunoresearch Cat# 703-545-155, RRID: AB_2340375). In NeuroGT database, the binding of anti-βGAL antibodies was enzymatically visualized with DAB. For that, the sections were treated with 0.7% H_2O_2/ methanol for 5 min at 4°C after antigen retrieval and incubated with the anti-βGAL primary antibody, donkey biotin-SP-conjugated anti-chicken IgY (1:1000, Jackson Immunoresearch Cat# 703-065-155, RRID: AB_2313596), and the Elite ABC kit, as described above.

For the double immunostaining of cortical neurons (Figures S1A and S1B), antigen-retrieved sections were incubated with chicken anti-βGAL antibody together with rat monoclonal anti-CTIP2 (1:1000, Abcam, Cat# ab18465, RRID: AB_2064130) or mouse monoclonal anti-SATB2 (1:1000, Abcam, Cat# ab51502, RRID: AB_882455) antibody, and then stained with donkey Alexa488-conjugated anti-chicken IgY and donkey Cy3-conjugated anti-rat IgG (1:1000, Jackson Immunoresearch Cat# 712-166-153, RRID:AB_2340669) or donkey Cy3-conjugated anti-mouse IgG (1:1000, Jackson Immunoresearch Cat# 715-165-150, RRID:AB_2340813) antibodies. In Figure S1C, to minimize cross-reactions of the anti-rabbit secondary antibody, sections were first stained with rabbit anti-GABA (1:2000, Sigma-Aldrich, Cat# A2052, RRID: AB_477652) and donkey Cy3-conjugated anti-rabbit IgG (1:1000, Jackson Immunoresearch Cat# 711-165-152, RRID: AB_2307443) antibodies, and then visualized for βGAL as described. Rabbit anti-NRP1 antibody was prepared and used as previously described (1:2000, Kawakami et al., 1996; Sato et al., 1998). In Figures S1E and S1G, sections were immunostained for mGFP with a chicken anti-GFP (1:1000; Abcam Cat# ab13970, RRID: AB_300798) and anti-chicken IgY antibodies, after staining for NRP1 as described above. The fluorescent samples were counterstained with DAPI (4',6-diamidino-2-phenylindole, 045-30361, CAS#28718-90-3, Fujifilm Wako).

Image acquisition

Whole-mount brain images (Figure 2) were captured with a digital camera (EOS 6D, Canon) mounted on a dissection microscope (SZ61, Olympus). The sections (Figures 4A, 4B, 5A, 5B, 6A, 7A, 7B, S2A, and S2C) were imaged with a CCD camera (Olympus DP71) attached to a conventional fluorescent microscope (Zeiss Axioplan2) using Cell Sens Standard software. Wide tile images were constructed from multiple images using the Photomerge tool in Photoshop software (Adobe). In some cases (Figures 4C–4F and S1), fluorescent images were captured with an inverted confocal microscope (Olympus IX81 FV1000) using the Fluoview software (FV-ASW ver4.02). Wide images were automatically constructed via multi-area time-lapse processing with a built-in mosaic imaging tool. The transmission images in Figures 6B–6D, S2B, and S2D were cropped from the digital images prepared for the NeuroGT database, for which the imaging procedures are explained in the following section. All the obtained images were rotated and cropped, and the brightness and contrast were non-linearly and equally adjusted across the entire image using Photoshop software (Adobe).

EdU incorporation assay

A thymidine analog, 5-ethynyl-2'-deoxyuridine (EdU, Tokyo Chemical Industry) was intraperitoneally injected into a pregnant mouse (50 mg/kg body weight) at the indicated time points before or after TM injection. The brains were dissected from the mice at P0, and coronal sections of 16-μm thickness were prepared as explained above. The sections were antigen-retrieved as described above and immunostained with chicken anti-βGAL and donkey Alexa488-conjugated anti-chicken IgY antibodies. Subsequently, the incorporated EdU was detected in a solution of 0.1 M Tris (pH 7.6), 2 mM $CuSO_4$, 3 μM Alexa555 azide triethylammonium salt (A20012, Thermo Fisher Scientific), and 10 mM ascorbic acid for 40 min at room temperature. The numbers of neurons labeled for βGAL and those doubly labeled for βGal and EdU were manually counted for each mouse with a 40× objective lens (Plan-Apochromat) under a fluorescent microscope (Zeiss Axioplan2) with 38 HE (ex470/40, em525/50) and 15 (ex549/12, em590) filter sets. The exact numbers of animals and neurons used for quantification are shown in the figures and legends. The scattered plots (Figures 5C–5F and 7C–7F) were generated using the Prism8 software (GraphPad).

Construction of the NeuroGT database

The brains were collected from P7 mice that had been tagged on a single day during E9.5–E18.5 using each neurogenic tagging mouse driver. Whole brains were cut into serial coronal sections (20-μm thick) from anterior to posterior, and each section was pasted on a batch of 10 glass slides in rotation. From the batch, three interspaced slides were antibody stained for the mGFP reporter, and three others were antibody stained for the nls-βGAL reporter. The antibody reactions were enzymatically visualized using DAB, as described above. Slide-fixed sections were dehydrated and mounted using Entellan New (Merck). The staining procedures and antibodies were slightly modified for the samples, and all these details are provided as meta-information in the NeuroGT database.

Images of the glass slides containing the immunostained multiple sections were acquired with a slide scanner (SCN400, Leica) using its designated software (Leica SCN400 Client Version 2.2.0.3789). The contrast and brightness were adjusted automatically. The images acquired using a 20× objective lens were extracted and separated into individual images of single sections using the Fiji distribution package of ImageJ (RRID: SCR_002285; Schindelin et al., 2012). The images of individual sections were aligned along the antero-posterior axis of the brain by rotating at 180° if needed.

The NeuroGT database was established as a web-based system using the Django framework with the PostgreSQL database on a Linux container with the Nginx server. An interactive image viewer was constructed using JavaScript. To encourage the operation, the database was organized as SSBD (Tohsato et al., 2016) with common meta-information such as organism name and contact information of the corresponding author in a unified format. The meta-information specific to NeuroGT, including driver name, TM stage and reporter, has also been provided.

QUANTIFICATION AND STATISTICAL ANALYSIS

The exact numbers of animals and neurons used for quantification are shown in Figures 5C–5F and 7C–7F. The proportions of tagged neurons containing EdU were calculated using Excel for Mac (Microsoft) and the raw counting data were archived in SSBD:repository (https://doi.org/10.24631/ssbd.repos.2021.03.001). The scattered plots and means were generated using the GraphPad Prism 8. In this study, the purpose of quantification was to show a trend of the continuous developmental process but not to differentiate a specific developmental stage from the others; thus, dichotomous significance testing was not performed.

ADDITIONAL RESOURCES

All images acquired in this study are available in the NeuroGT database at https://ssbd.riken.jp/neurogt/. Raw images and other data are available in SSBD:repository (https://doi.org/10.24631/ssbd.repos.2021.03.001).

Cell Reports Methods, Volume *1*

Supplemental information

NeuroGT: A brain atlas of neurogenic tagging CreER drivers for birthdate-based classification and manipulation of mouse neurons

Tatsumi Hirata, Yukako Tohsato, Hiroya Itoga, Go Shioi, Hiroshi Kiyonari, Sanae Oka, Toshihiko Fujimori, and Shuichi Onami

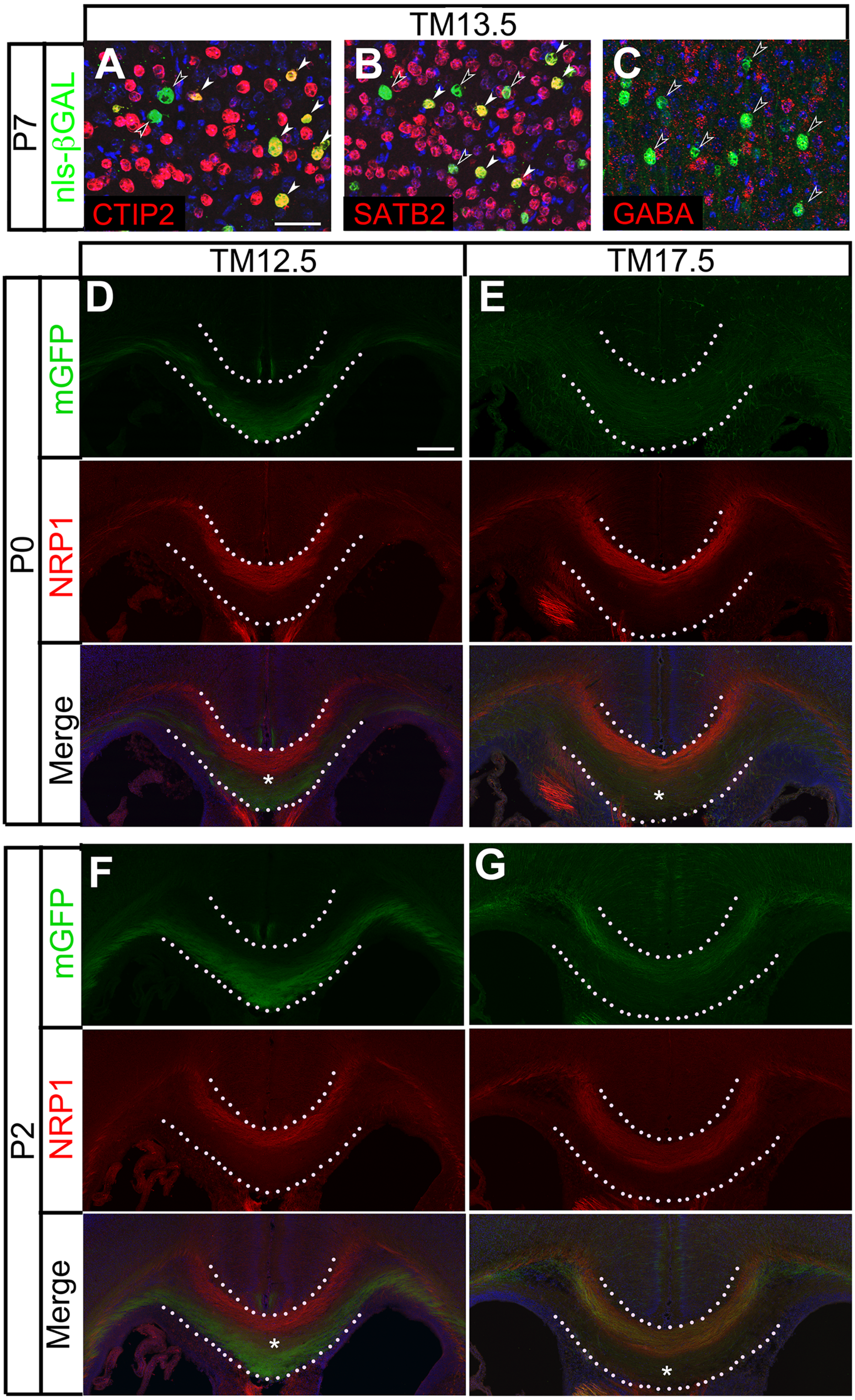

Figure S1. Cortical neurons and callosal projections tagged by Neurog2^{CreER}(G2A), Related to Figure 4
(A–C) Double immunostaining of TM13.5 cortical neurons in layer V for nls-βGAL (green) and anti-CTIP2 (red in A), anti-SATB2 (red in B) or anti-GABA (red in C) antibodies, and counterstained with DAPI (blue). Some tagged neurons express the subcortically projecting neuron marker CTIP2 (filled arrowheads in A) or callosally projecting neuron marker SATB2 (filled arrowheads in B), but not the inhibitory interneuron marker GABA (C). Open arrowheads indicate tagged neurons negative for these markers. (D–G) Corpus callosum sections (16-μm thick) obtained from P0 (D, E) and P2 (F, G) mice that were tagged at TM12.5 (D, F) or TM17.5 (E, G). Dotted lines delineate the corpus callosum. mGFP (green), anti-NRP1 (red) and their merged images counterstained with DAPI (blue). NRP1 marks the dorsal callosal bundle projected from the medial cortical areas only in the embryonic and neonatal stages. TM12.5-tagged axons course through the ventral edge of the callosum both at P0 (D) and P2 (F), leaving a small open space (asterisk) from the NRP1-positive bundle. TM17.5-tagged axons seem to still elongate during these neonatal stages; the axons are widely distributed across the whole callosum at P0 (E), whereas they are more concentrated in the dorsal zone marked by NRP1 and sparsely scattered in the ventral zone (asterisk) at P2 (G). For visualization of the TM17.5-tagged axons (E, G), an anti-GFP antibody was used because mGFP fluorescence was still weak at the neonatal stages. Bar= 200 μm.

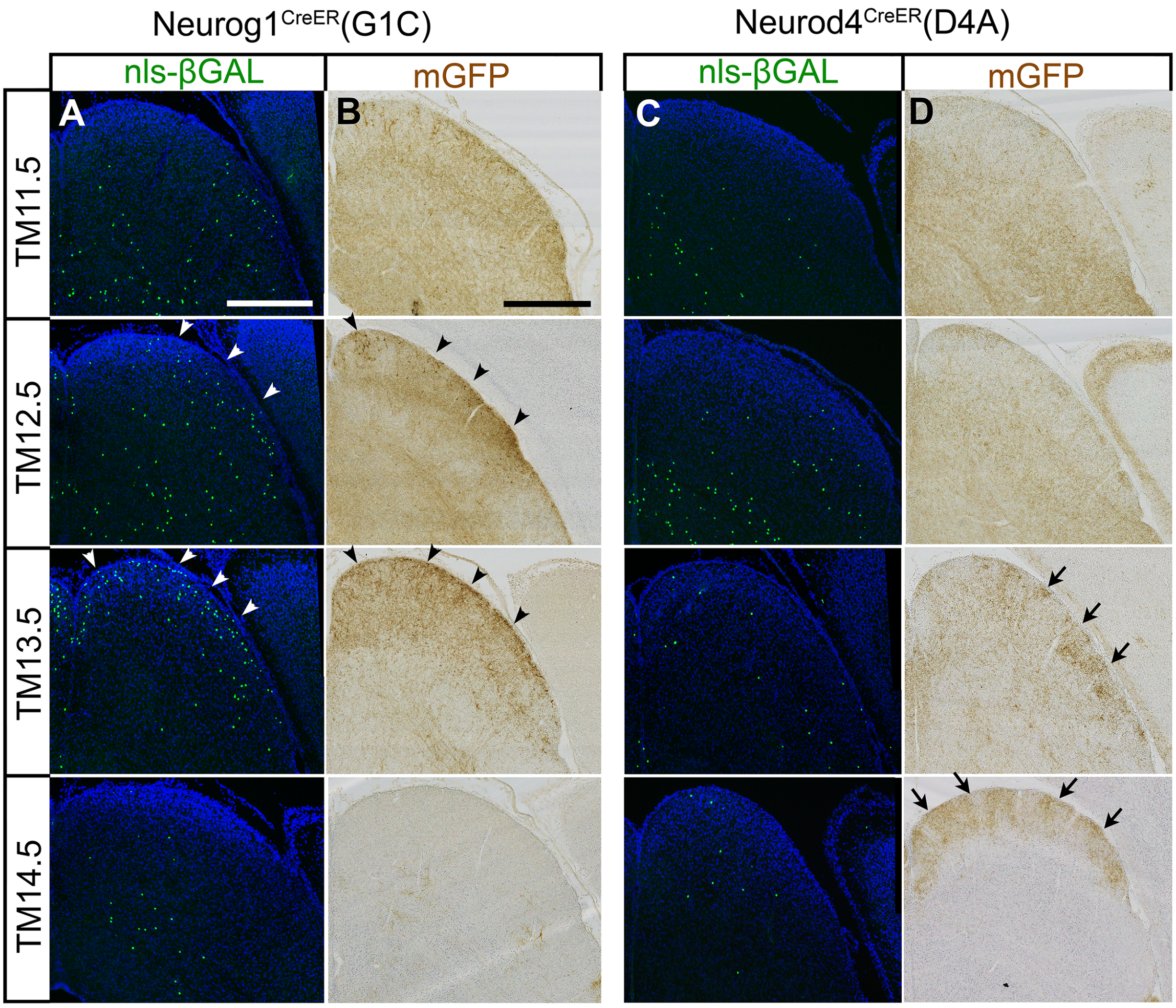

Figure S2. Superior colliculus tagged by Neurog1^{CreER}(G1C) and Neurod4^{CreER}(D4A), Related to Figure 6
(A–D) nls-βGAL (A, C) and mGFP (B, D) staining of superior colliculus sections obtained from P7 mice tagged by Neurog1^{CreER}(G1C) (A, B) and Neurod4^{CreER}(D4A) (C, D) drivers at different stages indicated on the left. Neurons in the superficial sensory layers (arrowheads) are labeled at TM12.5-13.5 by Neurog1^{CreER}(G1C) driver (A, B). Arrows in (D) indicate tagged retinal axons that target the superficial layers. Bar= 500 μm.
